# Limited Correlation of Shotgun Metagenomics Following Host Depletion and Routine Diagnostics for Viruses and Bacteria in Low Concentrated Surrogate and Clinical Samples

**DOI:** 10.3389/fcimb.2018.00375

**Published:** 2018-10-23

**Authors:** Corinne P. Oechslin, Nicole Lenz, Nicole Liechti, Sarah Ryter, Philipp Agyeman, Rémy Bruggmann, Stephen L. Leib, Christian M. Beuret

**Affiliations:** ^1^Biology Division, Spiez Laboratory, Swiss Federal Office for Civil Protection, Spiez, Switzerland; ^2^Institute for Infectious Diseases, University of Bern, Bern, Switzerland; ^3^Graduate School for Cellular and Biomedical Sciences, University of Bern, Bern, Switzerland; ^4^Interfaculty Bioinformatics Unit and Swiss Institute of Bioinformatics, University of Bern, Bern, Switzerland; ^5^Infectious Diseases Division, Department of Paediatrics, University Hospital Bern, Bern, Switzerland

**Keywords:** central nervous system infection, CSF, diagnostics, viruses, bacteria, NGS, shotgun metagenomics, host depletion

## Abstract

The etiologic cause of encephalitis, meningitis or meningo-encephalitis is unknown in up to 70% of cases. Clinical shotgun metagenomics combined with host depletion is a promising technique to identify infectious etiologies of central nervous system (CNS) infections. We developed a straightforward eukaryotic host nucleic acid depletion method that preserves intact viruses and bacteria for subsequent shotgun metagenomics screening of clinical samples, focusing on cerebrospinal fluid (CSF). A surrogate CSF sample for a CNS infection paradigm was used to evaluate the proposed depletion method consisting of selective host cell lysis, followed by enzymatic degradation of the liberated genomic DNA for final depletion with paramagnetic beads. Extractives were subjected to reverse transcription, followed by whole genome amplification and next generation sequencing. The effectiveness of the host depletion method was demonstrated in surrogate CSF samples spiked with three 1:100 dilutions of Influenza A H3N2 virus (qPCR Ct-values 20.7, 28.8, >42/negative). Compared to the native samples, host depletion increased the amount of the virus subtype reads by factor 7127 and 132, respectively, while in the qPCR negative sample zero vs. 31 (1.4E-4 %) virus subtype reads were detected (native vs. depleted). The workflow was applied to thirteen CSF samples of patients with meningo-/encephalitis (two bacterial, eleven viral etiologies), a serum of an Andes virus infection and a nose swab of a common cold patient. Unlike surrogate samples, host depletion of the thirteen human CSF samples and the nose swab did not result in more reads indicating presence of damaged pathogens due to, e.g., host immune response. Nevertheless, previously diagnosed pathogens in the human CSF samples (six viruses, two bacteria), the serum, and the nose swab (Human rhinovirus A31) were detected in the depleted and/or the native samples. Unbiased evaluation of the taxonomic profiles supported the diagnosed pathogen in two native CSF samples and the native and depleted serum and nose swab, while detecting various contaminations that interfered with pathogen identification at low concentration levels. In summary, damaged pathogens and contaminations complicated analysis and interpretation of clinical shotgun metagenomics data. Still, proper consideration of these issues may enable future application of metagenomics for clinical diagnostics.

## Introduction

Various known pathogens, including viruses, bacteria, fungi and parasites, cause severe infections of the central nervous system (CNS) accounting for 30–50% of cases of meningitis, encephalitis, and meningo-encephalitis (Glaser et al., 2006; Mailles and Stahl, 2009; Granerod et al., 2010). Mortality can be high as observed with the DNA virus *Herpes simplex* (treated 25%, untreated 70%), which causes the most lethal viral CNS infections endemic in the USA (George et al., 2014; Whitley, 2015). The etiology of meningo-/encephalitis cannot be identified in up to 70% of the total cases (Cizman and Jazbec, 1993; Sivertsen and Christensen, 1996; Khetsuriani et al., 2002; Glaser et al., 2006). Diagnosis of CNS infections, which is routinely performed in cerebrospinal fluid (CSF), remains a great challenge. Patients are usually in a severe state of health, thus broad-spectrum antibiotics and antivirals are frequently administered prior to CSF sampling affecting the diagnostic outcome. Moreover, long lasting routine liquor cultivation remains difficult as the majority of species are non-cultivable. Finally, fast routine molecular diagnostics by polymerase chain reaction (PCR) is restricted to the detection of known infectious agents.

For adequate patient management, improved identification of CNS infection etiologies to enable targeted therapy is indispensable. Metagenomics was recently used to assist infectious disease diagnostics, also for meningo-/encephalitis (Cordey et al., 2014; Naccache et al., 2015; Ortiz Alcántara et al., 2015; Kawada et al., 2016; Wylie et al., 2016; Lewandowska et al., 2017; Ruppé et al., 2017). Shotgun metagenomics, that sequences all nucleic acids (NA) in a given sample, has the potential to identify known but mutated (evading qPCR detection), unexpected, and previously undetected pathogens or mixed infections (Palacios et al., 2008; Basmaci et al., 2011; Smits et al., 2013; Tan le et al., 2013). Nonetheless, shotgun metagenomics has to deal with the vast proportion of host sequences in clinical samples. Existing host depletion approaches to enrich pathogen sequences are time consuming, expensive and/or require high specialization (Allander et al., 2001; Kapoor et al., 2008; Victoria et al., 2008; Ng et al., 2009; Kohl et al., 2015). They focus on either bacterial or viral DNA, or are only based on RNA sequencing targeting bacterial 16S or viral RNA (Allander et al., 2001; Hall et al., 2014; Jensen et al., 2015; Kohl et al., 2015; Lewandowska et al., 2015; Ruppé et al., 2017; Sabat et al., 2017).

This study aimed to establish a straightforward shotgun metagenomics workflow to simultaneously screen for both, bacteria and viruses in liquid clinical samples, focusing on human CSF. A simple host nucleic acid depletion method was developed to minimize the overwhelming host NA proportion, thus enriching presumed viral and bacterial NA within a patient's sample. Surrogate CSF samples were developed to model inflammatory CSF of patients with meningo-/encephalitis to validate the shotgun metagenomics workflow comparing depleted to native aliquots. The novel approach was applied to clinical CSF samples, a human nose swab and a serum sample.

The proposed host NA depletion method successfully increased viral and bacterial reads in surrogate CSF samples, which was not reproducible with clinical samples. The previously diagnosed pathogens were detected by shotgun metagenomics in the majority of samples but evident etiology was affected by low concentrations of pathogens, numerous contaminations and curation and extent of reference databases for bioinformatics analysis. Nonetheless, our shotgun metagenomics workflow is a novel approach to simultaneously detect RNA and DNA viruses as well as bacteria applying a total host nucleic acid depletion method.

## Materials and methods

### Samples

Native surrogate samples, samples of patients and negative controls were left unprocessed at room temperature during sample preparation until NA extraction.

#### Surrogate CSF samples (surrogCSF)

Immortalized cell lines were used to model a surrogate inflammatory CSF of a CNS infection. Human hepato-cellular carcinoma cells (HUH7; kindly provided by Dr. Volker Thiel at the Institute of Virology and Immunology, University of Bern) resemble active immune cells during the immune response (Shay et al., 2001). The cells were cultured in liquid Dulbecco's Modified Eagle Medium (DMEM) with stable glutamine (3.7 g/l NaHCO_3_ and 4.5 g/l D-glucose) supplemented with 0.1X DMEM volume of fetal bovine serum (FBS Superior), 0.01X non-essential amino acids 100x concentrate, 0.02X 1 M 4-(2-hydroxyethyl)-1-piperazineethanesulfonic acid (HEPES) buffer 50x, 0.01X Penicillin/Streptomycin liquid 10,000 μg/ml (Biochrom, Merck KGaA), and 0.00005X Neomycin sulfate 10 mg/ml (Pan—Biotech GmbH). The cells were split once before harvesting in culture medium without antibiotics. After harvesting and centrifugation, the cells were resuspended in artificial CSF (ASCF, Tocris® Bioscience) to a concentration mimicking a strong immune response (10,000 cells per μl) (Solomon et al., 2007). Aliquots of 50 μl in DNA LoBind 1.5 ml tubes (Eppendorf AG) were frozen at −80°C for stock. Cell suspensions were spiked with viral and/or bacterial suspension(s) to appropriate high concentrations well-detectable by qPCR in native and host NA depleted samples to a total volume of 60 μl [average Ct-values in native samples: viruses Ct-value 21.5 and bacteria 18, corresponding to copy number equivalents per 60 μl sample of 3.2E+6 viruses and 1.2E+8 bacteria copies, values higher or similar compared to other approaches (Cheval et al., 2011; Hasan et al., 2016)].

#### Pathogens

To test the physical influence of sample processing steps on human cells, bacteria, and viruses based on their cell/particle size, genome size and type, we chose a Gram-negative aerobe bacterium and an enveloped segmented RNA-virus of biosafety level 2 for ease of handling and higher susceptibility to physical homogenization, e.g., than Gram-positive bacteria or non-enveloped viruses. Thus, surrogCSF were spiked with the Gram-negative species *Yersinia pseudotuberculosis* (*Y.pseud*.) as a bacterial model (Nr. 1.5, Istituto cantonale di microbiologia Bellinzona, L'Ente Ospedaliero Cantonale, EOC) cultured overnight at 37°C on tryptic soy agar plate. The enveloped segmented (-)ssRNA Influenza A H3N2 virus (Inf A) was used as a viral model (Influenza A virus/Wisconsin/67/05 Nr. 13/8525.02.07/NCPV H3N2; P1, MDCK-cells, stored at −80°C).

#### Ethics statement

This study was carried out in accordance with the Declaration of Helsinki, the Human Research Act, and the Human Research Ordinance of the Swiss confederation. The protocol was approved by the ethics committee of the canton of Bern [Ref.-Nr: KEK 234/2014]. For the use of CSF samples from subjects in Bern, the ethics committee of the canton of Bern waived the need for informed consent; all other subjects gave written informed consent in accordance with the Declaration of Helsinki.

#### CSF samples of patients (patCSF)

CSF samples of patients from different years and two hospitals were tested as follows: (i) retrospectively compiled CSF samples of patients with suspected meningo-/encephalitis, hospitalized at the University Hospital Bern between January 2011 and December 2012. (ii) CSF of patients with suspected meningitis/meningo-/encephalitis submitted to diagnostics at the Institute for Infectious Diseases at the University of Bern in the year 2016 and 2017. The patCSF samples underwent accredited diagnostics (*i.a*. qPCR, serology, optical tests, and cultures) and the leftovers were frozen at −80°C. A patCSF declared negative after extensive routine analysis was used as negative patCSF sample. iii) CSF of patients with suspected meningitis/meningo-/encephalitis collected at the University Hospital of Basel in the year 2017. In contrast to the samples of i) and ii) the samples were immediately aliquoted for accredited classic diagnostics and metagenomic analysis, the latter stored up to one week at 4°C before freezing at −80°C. Classic diagnostics was performed using the FILMARRAY® Meningitis/Encephalitis (ME) Panel. Of all samples, those with the highest titer of the diagnosed pathogen were included in this study. Apart from diagnostics results patient's data were masked for all CSF samples presented in this study. Preceding the tests, the patCSF samples were thawed on ice and aliquoted in 70 μl samples into DNA LoBind 1.5 ml tubes (Eppendorf AG).

#### Serum sample

Peripheral blood was collected from a Swiss patient returning from South America (www.promedmail.org/; Archive Number: 20170118.4773312) via sterile venipuncture in tubes (Vacutainer®, Becton, Dickinson, and Company) at the University Hospital Bern. For the production of serum, tubes were centrifuged for 10 min at 1,000 g and serum was stored at −80°C (Kuenzli et al., 2018).

#### Nose swab sample

Nose swabs of a volunteer suffering from common cold were taken late in disease state. The swabs were immediately immersed in 500 μl Dulbecco's Phosphate Buffered Saline (PBS w/o CaCl_2_ & MgCl_2_, sterile-filtered, Merck KGaA) in DNA LoBind 1.5 ml tubes (Eppendorf AG) and put on ice. Before taking aliquots for analyses the tubes with the immersed swabs were vortexed for 1 min at highest velocity.

#### Negative controls

Negative controls were included in NGS analysis to control for possible contaminations in samples. Negative controls consisted of Dulbecco's Phosphate Buffered Saline (PBS w/o CaCl_2_ & MgCl_2_, sterile-filtered, Merck KGaA) or PCR grade water (Roche Diagnostics International AG). They were equally processed besides surrogate or human samples for the whole shotgun metagenomics workflow.

### Sample preparation

The host NA depletion method was developed to minimize the host reads proportion of NGS analysis and to shift the reads ratio to the presumed viral and bacterial pathogens within a patient's sample. A series of preliminary tests for method development was conducted, including different host cell lysis methods, free NA depletion methods, and NA extraction methods (Supplementary Datas 1.1, 1.2). The method starts with selectively lysing the host cells using a bead-beater Precellys™24 tissue homogenizer with a Soft tissue homogenizing Lysing Kit (CK14−0.5 mL, Bertin Corp., CNIM). The settings were 6,200 rpm for 20 s repeated 3 times with 10 s pause in-between. The Precellys™ tubes were then centrifuged for 60 s at 10,000 g and the whole sample volume was transferred to a new DNA LoBind 1.5 ml tube (Eppendorf AG). In order to increase capture efficiency of NA by paramagnetic beads, an enzymatic degradation was performed. To degrade released eukaryotic NA, 0.5 μl Benzonase® nuclease (Sigma-Aldrich®, Merck KGaA) and 3.5 μl 1 M MgSO_4_ (Sigma-Aldrich®, Merck KGaA) were added and incubated on a thermal shaker for 30 min at 37°C and 500 rpm. To deplete resulting NA fragments, 1.8X sample volume of AMPure® XP beads suspension (Agencourt®, Beckman Coulter, Inc.) was added and mixed by pulse-vortex. The mixture was pulse-spun, incubated at room temperature for 15 min, pulse-spun again and placed on a magnetic rack for 3 min. The supernatant was transferred to a new DNA LoBind tube for subsequent NA extraction by the MagNA Pure 96 system with the DNA and Viral NA Large Volume Kit and Viral NA Universal LV 1000 3.0.1 run protocol (500 or 1,000 μl input, 50 or 100 μl elution volume) (Roche Diagnostics International AG).

### qPCR analysis

Copy number of host and spiked pathogen NA in native surrogCSF and its change with host NA depletion were estimated using qPCR. The primer systems used were ACTB human (Hs01060665_g1) and Eukaryotic 18S rRNA Endogenous Control (FAM™/MGB probe, non-primer limited) (Thermo Fisher Scientific Inc.), the mitochondrial NADH dehydrogenase subunit 1 (ND1) primer-probe set by He et al. (2002), the validated in-house primer-probe set InfAM (M-protein) for Inf A, and the validated in-house primer-probe set entF3 for *Y. pseud*. The qPCR analyses were performed on a LightCycler® 96 System (Roche Diagnostics International AG) using the TaqMan™ Fast Advanced Master Mix for DNA and TaqMan® Fast Virus 1-Step Master Mix for RNA analysis (Applied Biosystems™, Thermo Fisher Scientific Inc.). The run protocols of the respective master mixes for 45 cycles were: Fast Virus 1-Step: 300 s at 50°C, 20 s at 95°C, 45x (3 s at 95°C, 30 s at 60°C), Fast Advanced: 20 s at 95°C, 45x (3 s at 95°C, 30 s at 60°C). Negative qPCR results in host NA depletion evaluations were set to a Ct-value 42 for calculation purposes. Copy numbers are estimated given a Ct-value of 21 corresponding to a copy number of 10E+5 (slope of −3.33/log).

### Next generation sequencing (NGS)

#### Fragment library preparation for shotgun metagenomics

To be non-selective for DNA-/RNA-viruses and bacteria, extractives were reverse transcribed and amplified. The protocol combined the reverse transcription (RT) kit SuperScript™ III First-Strand Synthesis SuperMix (Invitrogen™, Thermo Fisher Scientific Inc.) and the whole genome amplification (WGA) kit PicoPLEX® WGA Kit (Rubicon Genomics Inc., Takara Bio Inc.) (applied method if nothing else is stated). Therefore, the manufacturer's protocol for SuperScript™ III was altered and run for 5 min at 75°C for the random hexamers primer annealing, followed by 10 min at 25°C and 50 min at 55°C for RT. The RT product was diluted to 50 pg/μl of which, 1 μl diluted RT product was mixed with 4 μl of Cell Extraction Buffer of the PicoPLEX™ WGA kit and then proceeded according to manufacturer's protocol using 16 amplification cycles. To evaluate the potential benefit of a selective RNA amplification, the Complete Whole Transcriptome Amplification Kit (WTA2, Sigma-Aldrich®, Merck KGaA) was used with a large input volume. 18 μl of extractives were mixed with 2.5 μl Library Synthesis Solution and 2.5 μl Library Synthesis Buffer, incubated for 5 min at 70°C and cooled down to 18°C. Then, 2 μl Library Synthesis Enzyme were added and incubated according to the manufacturer's protocol for the subsequent amplification reaction.

Amplification products of both kits were purified using the QIAquick PCR Purification Kit (Qiagen N.V.) according to the manufacturer's protocol with some modifications as previously published (Oechslin et al., 2017). During the entire workflow, DNA concentrations were measured using the Qubit® dsDNA High Sensitivity Assay Kit on the Qubit® 2.0 Fluorometer (Thermo Fisher Scientific Inc.) according to manufacturer's protocol.

#### Ion torrent™ sequencing

In-house Ion Torrent™ sequencing was performed. Libraries were prepared according to the Ion Xpress™ Plus and Ion Plus Library Preparation for the AB Library Builder™ System User Guide using the Ion Plus Fragment Library Kit (Thermo Fisher Scientific Inc.).

##### NA shearing

NA shearing of purified amplification products was performed on a Covaris® M220 System according to the Appendix A of the above mentioned guide. The shearing duration was adapted for the PicoPLEX® and the WTA2 products to 250 s for a final 200 bp sequencing library.

##### Size selection

Size selection of the adapter-ligated fragment library was performed using the ready-to-use Agencourt® AMPure® XP reagent (Beckman Coulter Eurocenter S.A.) as previously published (Oechslin et al., 2017). In brief, sequencing libraries were mixed with 0.5X (400 bp) or 0.8X (200 bp) sample volume of bead suspension and incubated at room temperature for 5 min. Sample was then placed on a magnetic rack (DynaMag™-2 magnet, Thermo Fisher Scientific Inc.) for 3 min until cleared supernatant formed. The supernatant was transferred to a new tube and mixed with 1.8X sample volume of bead suspension. The tube was processed as described above, except that supernatant was discarded. The pellet was washed twice by adding 500 μl of 70% ethanol, keeping it on the magnetic rack and rotating 4 times gently. Ethanol was removed and the bead pellet was air-dried for maximum 5 min. The pellet was re-suspended in 20 μl of Low EDTA TE buffer (1X TE buffer, 10 mM Tris-HCL, pH 8.0, 0.1 mM EDTA, Thermo Fisher Scientific Inc.) preheated to 39°C and incubated for 2 min at room temperature before putting the tube back on the magnetic rack. The supernatant, as final size selected sequencing library, was eventually transferred to a new tube. A further size selection method used was the LabChip® XT fractionation system (Caliper Life Sciences, Inc.) with the LabChip® XT DNA 750 Assay Kit according to manufacturer's protocol with the following settings: Extraction mode: Size Range (400 bp: 280 bp ± 20%, 200 bp: 314 bp ± 5%) and channel operation mode: eXtract and Stop (specified per sample in Supplementary Table 1).

Size distributions of sheared DNA fragments or size selected libraries were assessed using the Agilent 2100 Bioanalyzer system with the Agilent High Sensitivity DNA Kit (Agilent Technologies, Inc.).

Ion Torrent™ sequencing was performed on an Ion Proton™ and an Ion S5™ System using an Ion PI™ Chip Kit v3 and, respectively Ion 530™ (400 bp) or Ion 540™ (200 bp) Chip Kits. Sequencing template preparation was done using the Ion Chef™ Instrument and the Ion S5™ Calibration Standard. Sequencing output data are listed in Supplementary Table 1.

#### Illumina® sequencing

Illumina® sequencing was used to reach higher sequencing depths as with Ion Torrent™ technology. The purified amplification product was sent for library preparation and sequencing to the Functional Genomics Center Zürich (FGCZ). The TrueSeq Nano DNA libraries were run on a HiSeq 2500 V4, paired-end, read length 125 bp 350 bp insert size. Sequencing output data are listed in Supplementary Table 1.

### Sequencing analysis

#### Quality filtering and trimming, adapter trimming

Quality of datasets was checked using FastQC version 0.11.5 (www.bioinformatics.babraham.ac.uk/projects/fastqc), including amplification adapters (Ward and Heuermann, 2007; Kamberov et al., 2010). Quality trimming and filtering was performed using Trimmomatic version 0.33 (minimum read length of 31 bp) (Bolger et al., 2014). Adapter trimming of Ion Torrent™ data was performed by Trimmomatic while adapter trimming of Illumina® data was performed separately before running Trimmomatic using Cutadapt 1.9.2 and 1.14 (Martin, 2011) with the options –*times 5 –pair-filter* = *both* and an adapter list containing the respective amplification as well as the sequencing adapters (Ward and Heuermann, 2007; Kamberov et al., 2010; Martin, 2011). Trimming output data are listed in Supplementary Table 2.

#### Taxonomical classification

##### Kraken/BLAST®

Trimmed reads were taxonomically classified using Kraken version 0.10.5 beta (Wood and Salzberg, 2014). For classification, trimmed paired-end reads were merged to Kraken single-end read format using the read_merger.pl script of the Kraken package and catenated with the unpaired read output files of Trimmomatic. A custom database was built, since the default Kraken database (“Standard”) was not kept up to date (last update: December 2015) and did not contain a human reference genome. NCBI's EDirect (www.ncbi.nlm.nih.gov/books/NBK179288/) was used to download the reference sequences of a) the human assembly Genome Reference Consortium Human Build 38 patch release 10 (GRCh38.p10) (download: 22. March 2017), b) all viruses (“uncultured human fecal virus (viruses)” removed, GenBank assembly accessions: GCA_001857745.1, GCA_001857805.1, GCA_001857825.1) (download: 30. November 2016), c) all genomes of bacteria in complete status (download: 30. November 2016) and d) all genomes of bacteria in chromosome status (download: 2. December 2016) from the RefSeq (www.ncbi.nlm.nih.gov/refseq/) or Genbank (www.ncbi.nlm.nih.gov/genbank/) databases *(esearch -db assembly -query” “bacteria”[Organism] AND (latest[filter] AND “complete*+*genome”[filter] AND “chromosome*+*level”[filter] AND all[filter] NOT anomalous[filter])” | esummary | xtract -pattern DocumentSummary -if FtpPath_RefSeq -element FtpPath_RefSeq -else -element FtpPath_GenBank)*. The accession numbers of the compiled assemblies for the custom Kraken database are provided by the separate Supplementary Table 5 (Data Sheet 2). Previous to database building by Kraken the low complexity sequence regions of the reference sequences were masked to nucleotide ambiguity code N using DUST (Morgulis et al., 2006). The reduction of the false positive classifications to species (noise) was performed according to a previously published procedure (Oechslin et al., 2017). Taxonomical profiles were processed by viruses and bacteria individually. Reads not classified by Kraken were locally aligned to the NCBI's non-redundant nucleotide database (nt; ftp://ftp.ncbi.nlm.nih.gov/blast/db/nt.*) (download: 19. September 2017) using BLAST® 2.5.0 (Altschul et al., 1990) (*-max_target_seqs 1 -max_hsps 1*). The taxonomical classification rates per sample are listed in Supplementary Table 2.

##### Bowtie2

Additionally to the k-mer based classification by Kraken, sequencing reads were aligned to the respective representative reference genome of the diagnosed or spiked pathogen using Bowtie2 2.3.2 (Langmead and Salzberg, 2012) with the options *-X 800* and the pre-set options –*end-to-end –sensitive* for Ion Torrent™ and –*local –sensitve-local* for Illumina® data. Illumina® data were separately mapped as paired and unpaired data and the results were summarized. The reference sequences of the respective pathogens were downloaded from the RefSeq or Genbank databases and are listed in Supplementary Table 3.

## Results

### Evaluation of the host NA depletion method

**Figure 1:**
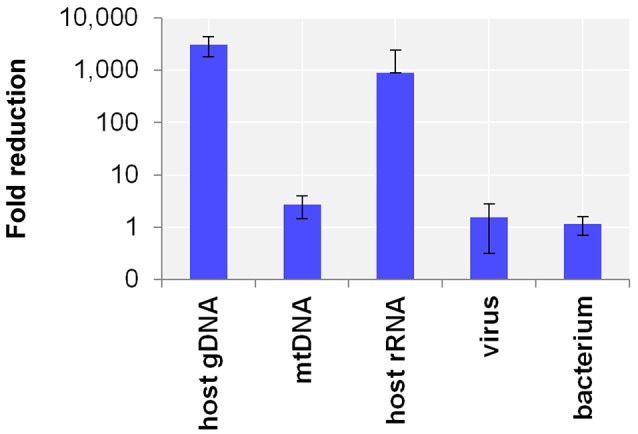
Evaluation of the host NA depletion method. Primer-probe targets listed on x-axis: the actin B gene host cell genomic DNA (gDNA), the mitochondrial NADH-ubiquinone oxidoreductase chain 1 DNA (mtDNA), the eukaryotic 18S ribosomal RNA (host rRNA), the matrix proteins of Influenza A virus (virus), the putative siderophore biosynthesis protein of *Y. pseudotuberculosis* (bacterium). Y-axis shows the net amount of depleted NA after host cell lysis as fold reduction in decimal logarithm scale comparing depleted to native samples of which the naturally free-floating NA were depleted previous to NA extraction using AMPure® XP beads reagent. Test was performed in 5 biological replicates and in 4 for bacteria. The error bars depict the standard deviation of the mean fold reduction of the replicates.

First, the efficacy of the host NA depletion method was evaluated by qPCR targeting host gDNA, mtDNA and rRNA as well as spiked bacteria and viruses comparing native to host NA depleted surrogCSF. Total depletion of host gDNA yielded a 280,000-fold reduction of host gDNA (±130,000 standard deviation; stddev; 5 replicates), mostly with end results being qPCR negative. The total average loss of virus was 340-fold (±540 stddev; 5 replicates) and of bacteria 14-fold (±10 stddev; 4 replicates). To assess the net amount of depleted NA after host cell lysis, host NA depleted surrogCSF were compared to a native sample, of which the naturally free-floating NA were depleted prior to NA extraction using the AMPure® XP paramagnetic beads reagent. The method yielded host gDNA net depletions of 3094-fold (±1275 stddev) with negligible net losses of virus and bacterium (1.5-fold with ±1.2 stddev and 1.2-fold with ±0.5 stddev, respectively) (Figure 1).

#### WTA for RNA viruses

**Figure 2:**
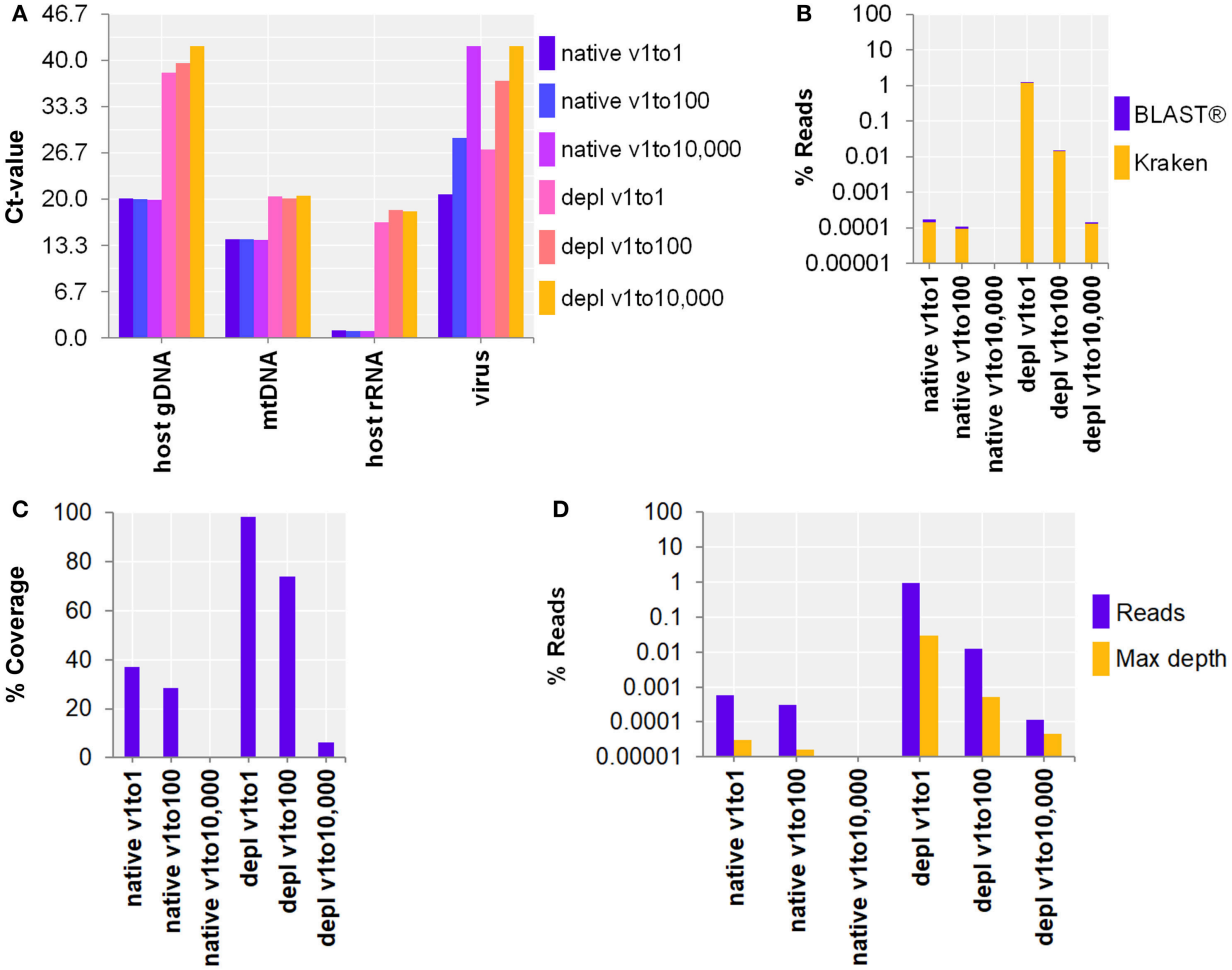
Whole transcriptome amplification kit (WTA2) for the metagenomic fragment library preparation for RNA virus spiked surrogate CSF samples with three 100-fold dilutions (v1to1, v1to100, v1to10,000) of Influenza A virus H3N2 virus (v; Inf A). **(A)** qPCR and **(B–D)** sequencing results of native and host NA depleted (depl) samples WTA2 amplified and NA extracted by automated MagNA Pure 96 system with the DNA and Viral NA Large Volume Kit and the Viral NA Universal LV 1000 3.0.1 run protocol. **(A)** Primer-probe targets listed on x-axis: the actin B gene host cell genomic DNA (host gDNA), the mitochondrial NADH-ubiquinone oxidoreductase chain 1 DNA (mtDNA), the eukaryotic 18S ribosomal RNA (host rRNA), the matrix proteins of Inf A H3N2 (virus). The y-axis shows the cycle threshold value (Ct-value) in steps of 3.33, representing an approximated 10-fold reduction of copy number. **(B)** Taxonomical classification of reads to Inf A H3N2 by exact k-mer mapping (Kraken) to a custom database of human, viruses and bacteria assemblies from RefSeq or Genbank complemented with local alignment to the NCBI's nt (BLAST®) of unclassified reads by Kraken. **(C)** % Reference sequence coverage and **(D)** reads aligned with maximum depth of coverage (Max depth) results of reference sequence alignments (Bowtie2) to Inf A H3N2. **(B–D)** Y-axis shows the number reads in % respective to the total reads that were taxonomically classified or assigned as unclassified by Kraken in decimal logarithm scale.

Next, the workflow for shotgun metagenomics was optimized to detect RNA viruses in CSF samples. SurrogCSF samples were spiked with three 100-fold dilutions of Influenza A virus H3N2 followed by sample preparation and NA extraction using the MagNA Pure 96 system (copy number estimates based on qPCR Ct-value: native with 3.7E+6, 1.4E+4, 1.5, and host NA depleted with 4.4E+4, 47, 1.5, respectively). The extractives were reverse transcribed and amplified using a whole transcriptome amplification kit. The qPCR revealed an effective depletion of host NA (host gDNA: 6, mtDNA: 2, host rRNA: 5 log cycles), but a loss of RNA virus (virus: 2–2.5 log cycles) compared to the native samples (Figure 2A). On the other hand, the Illumina® sequencing output analyzed by Kraken/BLAST® resulted in an increase of Inf A H3N2 reads by host NA depletion of 3.9 and 2.1 log cycles for the v1to1 and v1to100 samples, respectively. Besides reads assigned to spiked viruses, the vast majority of classified reads belonged to the host (in average 98.8%) and to numerous species likely derived from contaminations or reference database issues (Supplementary Table 4). Nevertheless, evaluation of the taxonomic profiles revealed the spiked virus species in all samples except of qPCR and sequencing negative native v1to10,000. Reference alignment showed similar values with 3.2 (v1to1) and 1.6 (v1to100) log cycles (Figures 2B–D). The lowest virus concentration (v1to10,000) was only detected within the host NA depleted surrogCSF with a few reads assigned to Inf A H3N2 (Kraken/BLAST®: 34 reads, 1.5E-4%, Bowtie2: 115 reads, 1.1E-4% with 6% reference coverage and 4.5E-5% maximum depth of coverage).

#### WGA for DNA viruses and bacteria

**Figure 3:**
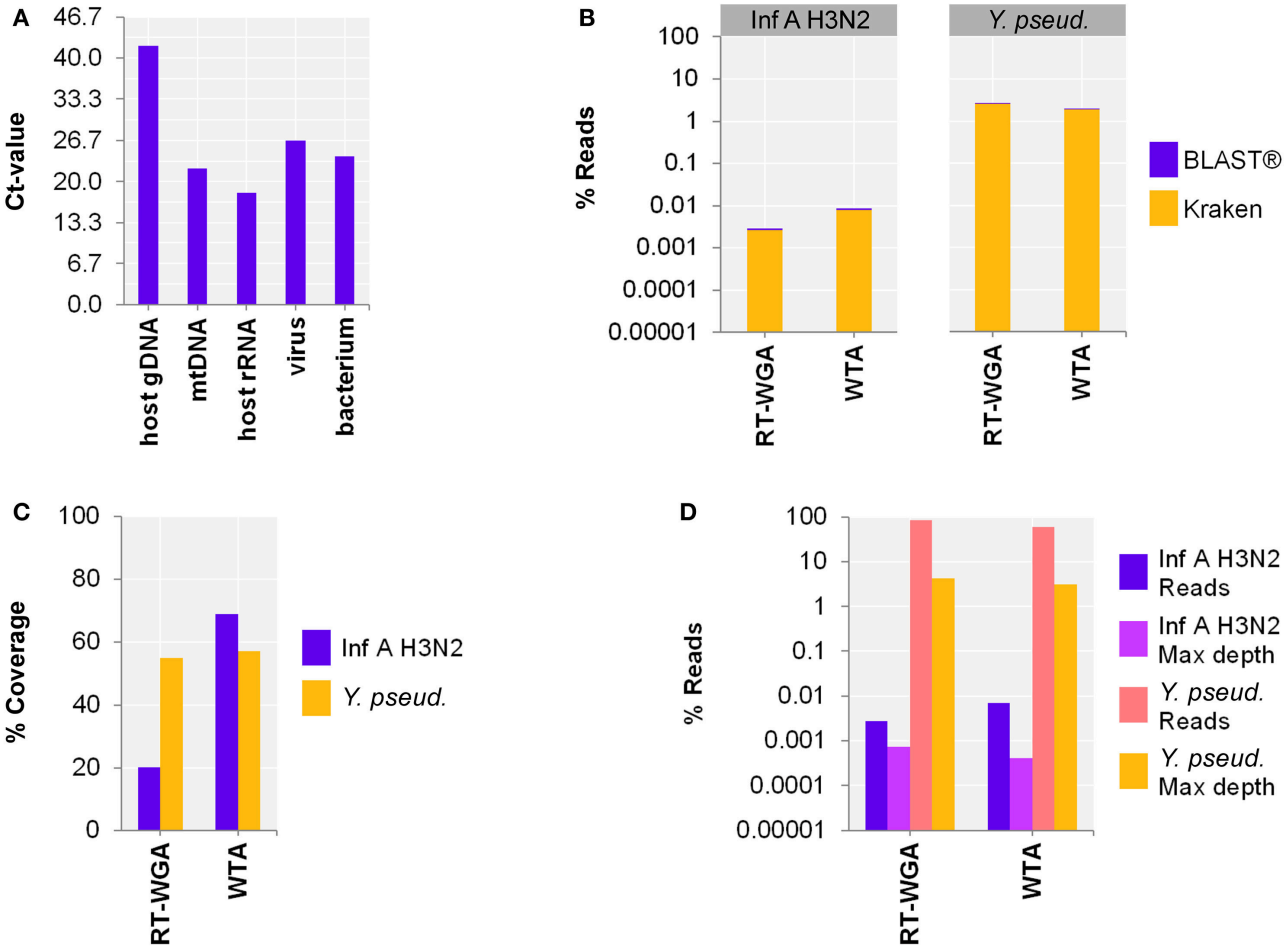
Comparing whole transcriptome amplification (WTA) to a reverse transcription and whole genome amplification (RT-WGA) approach for shotgun metagenomics analysis of a host NA depleted surrogate CSF sample spiked with RNA virus Influenza A virus H3N2 (Inf A) and bacteria *Y. pseudotuberculosis* (*Y. pseud*.). **(A)** qPCR and **(B–D)** sequencing results WTA2 compared to SuperScript™ III First-Strand Synthesis SuperMix followed by PicoPLEX® WGA Kit amplification systems for sequencing library preparation of NA extracted by MagNA Pure 96 with the DNA and Viral NA Large Volume Kit and Viral NA Universal LV 1000 3.0.1 run protocol. **(A)** Primer-probe targets listed on x-axis: the actin B gene host cell genomic DNA (host gDNA), the mitochondrial NADH-ubiquinone oxidoreductase chain 1 DNA (mtDNA), the eukaryotic 18S ribosomal RNA (host rRNA), the matrix proteins of Inf A (virus), the putative siderophore biosynthesis protein of (bacterium). The y-axis shows the cycle threshold value (Ct-value) in steps of 3.33, representing an approximated 10-fold reduction of copy number. **(B)** Taxonomical classification of reads to Inf A and *Y. pseud*., respectively, by exact k-mer mapping (Kraken) to a custom database of human, viruses, and bacteria assemblies from RefSeq or Genbank complemented with local alignment to the NCBI's nt (BLAST®) of unclassified reads by Kraken. **(C)** % Reference sequence coverage and **(D)** reads aligned with maximum depth of coverage (Max depth) results of reference sequence alignments (Bowtie2) to respective species. **(B,D)** Y-axis shows the number reads in % respective to the total reads that were taxonomically classified or assigned as unclassified by Kraken in decimal logarithm scale.

Since our aim was to simultaneously detect RNA and DNA, as a next step, we adjusted the protocol to include bacterial and viral DNA. Therefore, the use of a separate reverse transcription (RT; SuperScript™ III First-Strand Synthesis SuperMix) followed by whole genome amplification (WGA; PicoPLEX® WGA Kit) was compared to WTA using spiked host NA depleted surrogCSF (Figure 3A). The metagenomics analysis of the 2-step RT-WGA approach produced fewer RNA virus but slightly more bacterial reads compared to WTA (factor 3.1, 0.7, respectively) (Figure 3B). The reference coverage of both the RNA virus and the bacterium was lower after RT-WGA compared to WTA (factor 2.6, 0.7, respectively), along with a higher maximum depth of coverage after RT-WGA (factor 0.6, 0.7, respectively) (Figures 3C,D).

### Samples of patients

After successful implementation of the host NA depletion and bioinformatics analysis in surrogCSF, we validated the procedure with clinical samples. Of all patient samples, two aliquots were prepared, one as native and one as host NA depleted. Since the host NA depletion method presented with substantial depletion of host DNA, two-step RT-WGA was applied for amplification instead of WTA. Furthermore, WGA may amplified less host RNA compared to WTA, but included clinically important DNA viruses and supported detection of bacteria.

#### Clinical CSF samples

In patCSF samples, we detected the same pathogen as previously identified by routine diagnostics in eight of thirteen cases using our NGS approach in native and/or host NA depleted samples. A patCSF sample with a qPCR Ct-value 24 for Varicella-zoster virus (VZV) resulted in a positive NGS result with 0.0033% of reads classified by Kraken to the respective pathogen. Additionally, 1.2E-4% of the unclassified reads were assigned by BLAST® (273, 10 reads, respectively) (Table 1 sample 1, 2). Bowtie2 reference alignment assigned fewer reads to VZV compared to the Kraken/BLAST® analysis (252, 283 reads, respectively). PatCSF with low virus copy numbers (high Ct-values) ended up in just few reads taxonomically classified by Kraken/BLAST®, more specifically in native sample 3 (Ct-value 30.0, 1.7E-4% reads, 12 reads for HSV-1) and native sample 5 (Ct-value 35.5, 3.6E-5% reads, 3 reads for Enterovirus) of Table 1. No reads of all three host NA depleted patCSF were assigned to the respective pathogen. Bacteria positive samples detected by routine culture diagnostics resulted in more taxonomically classified reads for sample 12 and 13 (positive for group A beta-hemolytic Streptococci, e.g., *S. pyogenes;* native: 0.0037% reads, 4247 reads and host NA depleted: 9.2E-4% reads, 882 reads) but less reads for sample 22/23 (positive for *S. aureus;* native: 5.3E-5% reads, 14 reads / host NA depleted: 2.8E-5% reads, 7 reads). Further patCSF results are listed in Table 1. Unbiased evaluation of the metagenomics profiles supported the diagnosed pathogens in sample 1 and 3 by clearly pointing out the pathogen species, despite the overwhelming proportions of host classified reads (85.6 and 81.8%, respectively) and numerous species likely to originate from contaminations or erroneous reference sequences in the database.

**Table 1:** Overview of the routine diagnostics and NGS results of the sequenced human CSF samples, a nose swab and a serum sample.

**ID**	**Year**	**Routine diagnostics data**		**Prep**	**Kraken**		**BLAST**		**Bowtie2**				
												**Max depth of cov**		
					**%**	**Reads**	**%**	**Reads**	**%**	**Reads**	**% Cov**	**%**	**Reads**
**CSF**														
1	2011/2	VZV	ct 24.1	Native	0.0033	273	1.2E-4	10	0.0031	252	16.7	6.1E-5	5
2	2011/2	VZV	ct 24.1	Depl	0	0	0	0	0	0	0	0	0
3	2011/2	HSV-1	ct 30.0	Native	1.4E-4	10	2.8E-5	2	7.1E-5	5	0.5	1.4E-5	1
4	2011/2	HSV-1	ct 30.0	Depl	0	0	0	0	0	0	0	0	0
5	2011/2	Enterovirus	ct 35.5	Native	0	0	3.6E-5	3	0	0	0	0	0
6	2011/2	Enterovirus	ct 35.5	Depl	0	0	0	0	0	0	0	0	0
7	2011/2	Enterovirus	ct 30.6	Native	0	0	2.2E-5	1	0	0	0	0	0
8	2011/2	Enterovirus	ct 30.6	Depl	0	0	0	0	0	0	0	0	0
9	2016	Enterovirus	ct 27.9	Depl	0	0	0	0	0	0	0	0	0
10	2016	Enterovirus	ct 27.9	Native	0	0	0	0	0	0	0	0	0
11	2016	Enterovirus	ct 27.9	Depl	0	0	8.9E-6	6	0	0	0	0	0
12	2016	Group A beta- hemolytic Streptococci	culture/MALDI-TOF	Native	0.0035	4023	1.9E-4	224	0.038	43776	9.5	0.0024	2803	*1
					5.3E-5	61	2.6E-6	3	0.036	41955	4.9	0.0023	2687	*2
					5.3E-5	61	2.8E-5	32	0.034	39400	1.7	0.0025	2846	*3
					3.9E-5	45	1.7E-6	2	0.033	38497	1.9	0.0017	1961	*4
					4.1E-5	47	9.5E-6	11	0.034	39170	1.9	0.0017	1961	*5
13	2016	Group A beta- hemolytic Streptococci	culture/MALDI-TOF	Depl	8.6E-4	832	5.2E-5	50	0.032	30368	5.4	0.0022	2145	*1
					1.8E-5	18	6.2E-6	6	0.031	29865	3.9	0.0021	2060	*2
					6.5E-5	63	2.0E-5	19	0.030	29240	3.7	0.0022	2085	*3
					3.0E-5	29	0	0	0.030	28977	4.1	0.0016	1526	*4
					8.5E-5	82	1.8E-5	17	0.030	29347	4.1	0.0016	1526	*5
14	2016	JCV	120 cp/ml	Native	0	0	0	0	0	0	0	0	0
15	2016	JCV	120 cp/ml	Depl	0	0	0	0	0	0	0	0	0
16	2016	HSV	ct 27.0	Native	2.2E-6	1	0	0	4.3E-6	2	0.04	2.2E-6	1	*6
									2.2E-6	1	0.02	2.2E-6	1	*7
17	2016	HSV	ct 27.0	Depl	0	0	1.9E-6	1	0	0	0	0	0	*6
									7.6E-6	4	0.07	1.9E-6	1	*7
18	2016	VZV	ct 32.0	Native	0	0	0	0	4.7E-5	15	0.04	4.4E-5	14
19	2016	VZV	ct 32.0	Depl	0	0	0	0	9.8E-6	3	0.02	9.8E-6	3
20	2016	Enterovirus	ct 30.6	Native	0	0	0	0	0	0	0	0	0
21	2016	Enterovirus	ct 30.6	Depl	0	0	0	0	0	0	0	0	0
22	2016	*Staph. aureus*	culture/MALDI-TOF	Native	4.1E-5	11	1.1E-5	3	0.012	3213	0.5	7.9E-4	210
23	2016	*Staph. aureus*	culture/MALDI-TOF	Depl	2.0E-5	5	7.9E-6	2	0.0056	1424	0.4	3.7E-4	93
24	2017	Enterovirus	FILMARRAY®	Native	0	0	0	0	0	0	0	0	0
25	2017	Enterovirus	FILMARRAY®	Depl	0	0	0	0	0	0	0	0	0
26	2017	HHV-6	FILMARRAY®	Native	0	0	0	0	0	0	0	0	0
27	2017	HHV-6	FILMARRAY®	Depl	0	0	0	0	0	0	0	0	0
**NOSE SWAB**													
28	2017	Rhinovirus	ct 35.25	Native	7.8E-4	82	0.39	41085	2.9E-5	3	1.7	1.9E-5	2	*8
									3.8E-5	4	1.3	3.8E-5	4	*9
29	2017	Rhinovirus	ct 35.25	Depl	8.0E-5	9	0.068	7715	0	0	0	0	0
**SERUM**													
30	2016	ANDV	ct 27.0	Native	0	0	8.6E-4	40	0.0011	50	19.6	2.2E-4	10
31	2016	ANDV	ct 27.0	Depl	0	0	9.5E-4	74	3.8E-4	30	11.8	1.0E-4	8

Native and host NA depleted (depl) preparations of the same sample are listed. Read numbers of the different bioinformatics tools (Kraken, BLAST®, Bowtie2) refer to sequencing reads assigned to the respective pathogens previously detected by routine diagnostics (ct XX.XX corresponds to cycle threshold values of the respective qPCR evaluations). Samples 7–8 and 10–23 were sequenced on an Illumina® HiSeq 2500 V4 paired-end (samples 7–8: ^1^/_6_ lane, 10–23: ^1^/_2_ lane) and samples 1–6, 9, 24–31 on an Ion Torrent™ Ion S5™ using Ion 540™ Chips. Read numbers in % respective to the total reads that were taxonomically classified or assigned as unclassified by Kraken are listed. The reference genomes used for Bowtie2 analysis were either a reference or a representative genome of the NCBI's RefSeq database of the respective pathogen. For diagnosed infections by group A beta-hemolytic Streptococci the genomes were: S. pyogenes (^*^1), S. dysgalactiae subsp. equisimilis (^*^2), S. anginosus (^*^3), S. constellatus subsp. pharynges (^*^4), S. intermedius (^*^5); for Enterovirus: Human enterovirus A–D and Human rhinovirus A–C; for HSV: Herpes simplex virus 1 (^*^6), 2 (^*^7); for Rhinovirus: Human rhinovirus A (^*^8), B, C (^*^9); for HHV-6: Human herpesvirus 6, A, B. Prep, sample preparation; cov, reference coverage; cp/ml, copies per milliliter; ct, cycle threshold; VZV, Varicella zoster virus; JCV, JC polyomavirus; Staph. aureus, Staphylococcus aureus, ANDV, Andes virus.

#### Serum

To further investigate the applicability of our workflow to other sample sources, the serum of a patient with a severe Andes virus (ANDV, genus Orthohantavirus) infection was processed in a biosafety level 3 laboratory. ANDV was detected by routine qPCR diagnostics at a Ct-value of 27. After a second thawing step for subsequent NGS analysis, the control qPCR of the native sample was negative, whereas the virus was detected at Ct-value 35.5 in the host NA depleted sample. Kraken did not classify any reads to ANDV, but BLAST® assigned more reads to the host NA depleted serum compared to the native sample: 74 reads (9.5E-4%) and 40 reads (8.6E-4%), respectively. Unbiased evaluation of the taxonomic profile sustained the diagnosed pathogen, even though there was a high background signal (*i.a*. host classified reads of 88.7% and 47.3% of the native and the host NA depleted aliquot, respectively). A reference alignment (Bowtie2) showed a slightly better reference coverage (19.6, 11.8%) with more reads (50, 0.0011; 30 reads, 3.8E-4%) and accordingly a higher maximum depth of coverage (10, 2.2E-4; 8 reads, 1.0E-4%) of the native compared to the host NA depleted serum.

#### Nose swab

**Figure 4:**
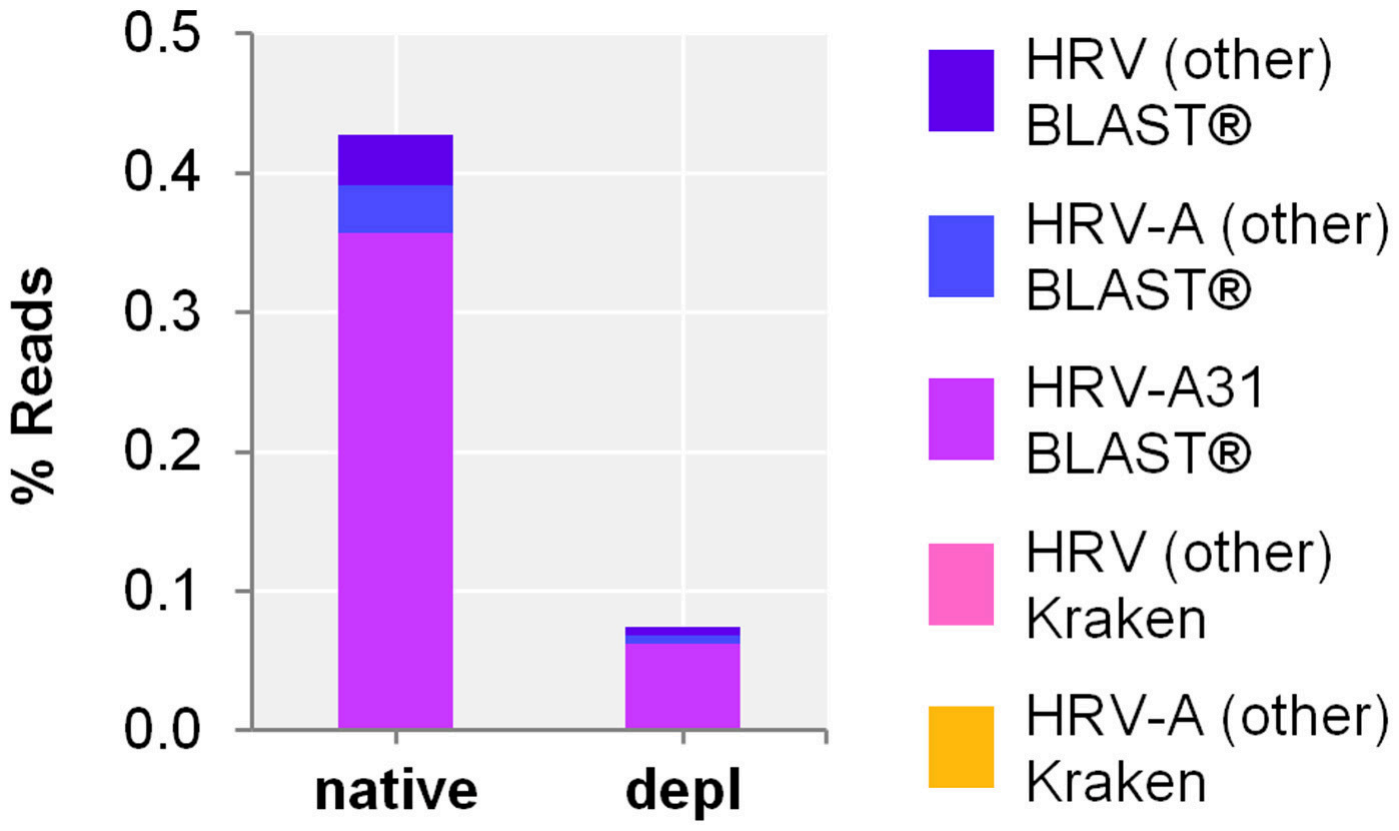
Nose swab sequencing results of previously defined Human rhinovirus (HRV) infection by qPCR. Taxonomical classification of reads to HRV-A, HRV-A31, and HRV other than HRV-A by exact k-mer mapping (Kraken) to a custom database of human, viruses and bacteria assemblies from RefSeq or Genbank complemented with local alignment to the NCBI's nt (BLAST®) of unclassified reads by Kraken. X-axis indicates the native and the host NA depleted (depl) preparation. Y-axis shows the number reads in % respective to the total reads that were taxonomically classified or assigned as unclassified by Kraken in decimal logarithm scale.

Host NA depletion and our NGS approach was furthermore applied to a nose swab taken from a volunteer on the fourth day of a common cold infection. The volunteer had already administered a flu nasal spray and took antiseptic and anesthetic troches for sore throat medication. The diagnosed Rhinovirus infection by qPCR (Ct-value 35.25) could be clearly confirmed by NGS through unbiased evaluation of the taxonomic profile of Kraken/BLAST® regardless of the high background signal (*i.a. Cutibacterium acnes* classified reads of 0.3 and 57.6% of the native and the host NA depleted aliquot, respectively). Kraken classified the Rhinovirus to Human rhinovirus A and specified it by BLAST(R) to serotype HRV-A31 (Figure 4) (Kraken: native: 44 reads, 4.2E-4%; host NA depleted: 8 reads, 7.1E-5%; BLAST®: native: HRV-A31: 0.4%, 37,282 of HRV-A: 40,837 and HRV: 41,085 reads; host NA depleted: 0.06%, 7,041 of HRV-A: 7,658 and HRV: 7,715 reads). Reference alignment (Bowtie2) resulted in few reads mapped for the native (3 reads, 2.9E-5%, coverage: 1.7%, maximum depth of coverage: 2 reads), whereas no read could be mapped of the host NA depleted sample to HRV-A.

### Negative controls, contamination, and noise

**Figure 5:**
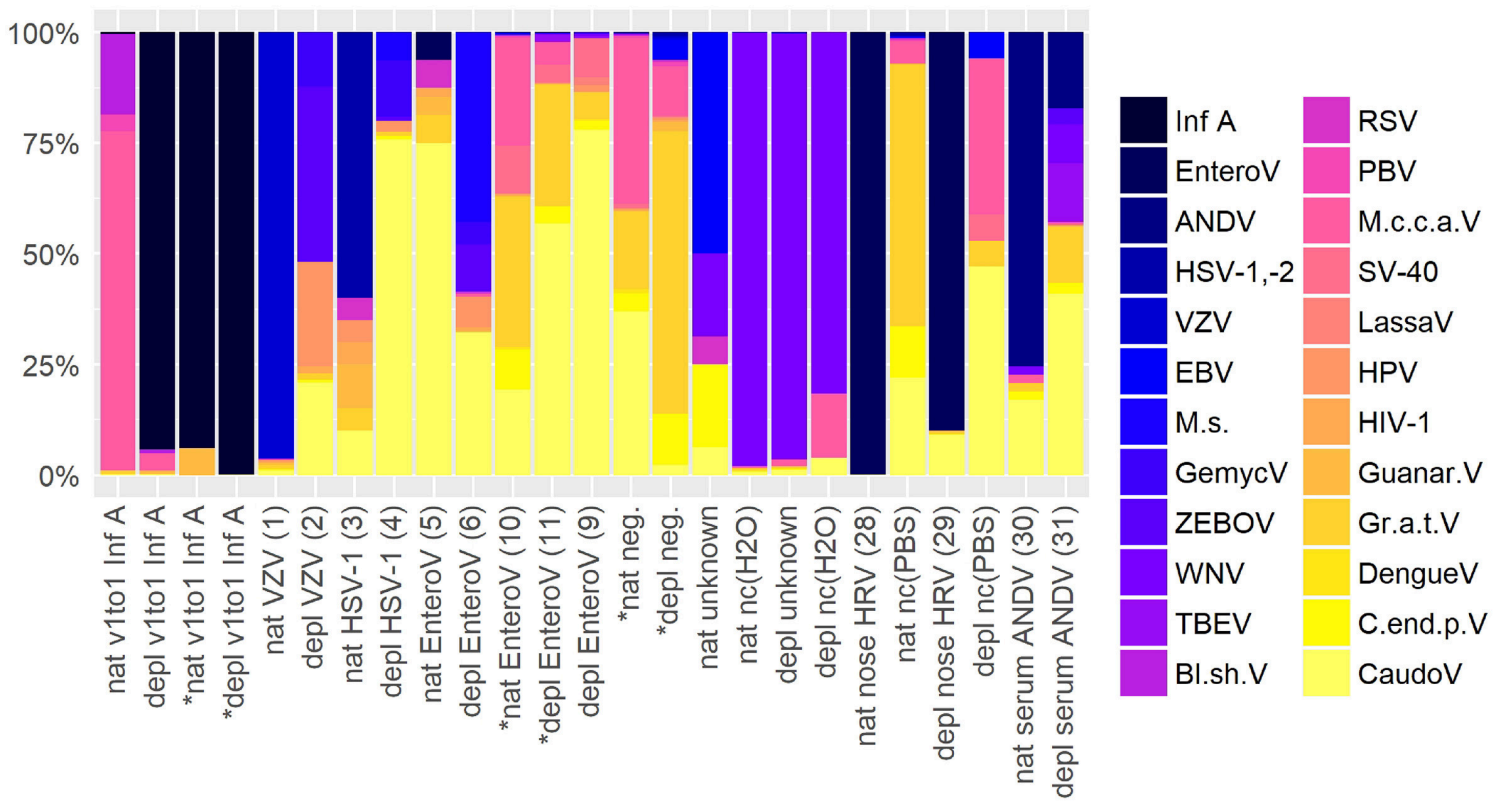
Abundance overview of most prominent virus species in shotgun metagenomics profiles in at least one across a set of different samples comprising native (nat) and host NA depleted (depl) surrogate CSF samples spiked with Influenza A virus (Inf A) and amplified using whole transcriptome amplification (WTA2) and samples of patients and negative controls (nc) amplified using sequential reverse transcription and whole genome amplification (SuperScript™ III First-Strand Synthesis SuperMix, PicoPLEX® WGA Kit) including CSF samples of patients the pathogen was detected in high (VZV) and low (HSV-1) amounts, diagnostically negative (neg.), and of unknown disease etiology accompanied by PCR grade water nc (H2O), furthermore a nose swab (nose HRV) accompanied by PBS nc, and a serum (serum ANDV). Majority of samples were sequenced on an Ion Torrent™ Ion S5™ using Ion 540™ Chips, few were sequenced on an Illumina® HiSeq 2500 V4 paired-end (^*^) on ^1^/_2_ lane (nat and depl EnteroV (samples 10, 11 of Table 1), nat and depl neg.), respectively on ^1^/_6_ lane (nat and depl v1to1 Inf A). HRV, Human rhinovirus; EnteroV, Enterovirus genus; ANDV, Andes virus; HSV-1,-2, Herpes simplex virus 1 and 2; VZV, Varicella zoster virus; EBV, Epstein-Barr virus; M.s., Mollivirus sibericum; GemycV, Gemycircularvirus genus; ZEBOV, Zaire ebolavirus; WNV, West Nile virus; TBEV, Tick-borne encephalitis virus; Bl.sh.V, Blueberry shoestring virus; RSV, Respiratory syncytial virus spp.; PBV, Picobirnavirus; M.c.c.a.V, Montastraea cavernosa colony-associated virus; SV-40, Macaca mulatta polyomavirus 1; LassaV, Lassa virus; HPV, Human papillomavirus spp.; HIV-1, Human immunodeficiency virus type 1; Guanar.V, Guanarito virus; Gr.a.t.V, Grapevine associated totivirus-1; DengueV, Dengue virus spp.; C.end.p.V, Citrus endogenous pararetrovirus; CaudoV, Caudovirales order (only Kraken results).

To assess contamination in human and surrogate samples detected by shotgun metagenomics, negative controls were included in the analysis. Figure 5 shows an abundance overview of the most prominent virus species in a set of twelve samples. Supplementary Figures 1A,B are the corresponding figures for bacteria and a merged view of fungi, parasites, plants, and vertebrates other than human. The listed samples comprise native and host NA depleted surrogCSF, diagnostically positive and negative samples of patients (patCSF, nose swab, serum) and negative controls. Including viruses and bacteria detected by routine diagnostics, the set of samples showed 24 virus, 30 bacterium, 3 fungus, 6 parasite, 2 plant, and 7 vertebrate species other than human dominating at least in one of the respective taxonomic profiles of summed reads of Kraken/BLAST® analysis.

Indeed, spiked Inf A virus in surrogCSF and previously identified pathogens by routine diagnostics in patCSF were taxonomically assigned in the respective samples, but Inf A was also detected with a few reads in the host NA depleted negative patCSF. Herpes simplex virus (HSV-1,-2) was additionally detected in Illumina® sequenced patCSF samples and within the native negative controls of water and PBS. Of the predominant viruses, tick-borne encephalitis virus (TBEV), West Nile virus (WNV) and Zaire Ebola virus (ZEBOV) could be identified as laboratory associated contaminants due to simultaneous processing of inactivated samples (reverse transcription, amplification) for Sanger and/or whole genome sequencing in the same laboratory room. Additionally detected Lassa, Guanarito, and Dengue virus by NGS were possibly laboratory associated contaminants due to the same reason as mentioned before, but could not be definitely confirmed by specific PCR. Other human pathogenic viruses including the Epstein-Barr virus (EBV), the Respiratory syncytial virus (RSV), the Picobirnavirus (PBV), the Human immunodeficiency virus type 1 (HIV-1), the Human papillomavirus (HPV) and the Macaca mulatta polyomavirus 1 (SV-40) were predominantly assigned in the viral taxonomic profiles in at least one of the human, the surrogCSF and the negative control samples. The unexpected Montastraea cavernosa colony-associated virus (M.c.c.a.V) was predominant in the native and (besides the spiked Inf A) the host NA depleted surrogCSF samples, sequenced on an Ion Torrent™ platform, but not in sample aliquots sequenced on an Illumina® platform. On the other hand, many reads were assigned to M.c.c.a.V in a native patCSF (sample 10 Table 1) and a native negative patCSF sequenced on an Illumina® platform. Further unexpected viruses were the Blueberry shoestring virus, the Citrus endogenous pararetrovirus, the Grapevine associated totivirus-1 and the Mollivirus sibericum.

Regarding bacteria, *Cutibacterium acnes* was excluded due to an overwhelming presence of 65% in average, except in the native v1to1 (2.4 and 0.4%, sequenced on a IonTorrent™ and an Illumina® platform, respectively) and native EnteroV (10) (6.4%) samples. While *Francisella tularensis* was identified as laboratory associated bacterial contaminant, *Borrelia/-ella* genera and *Coxiella burnettii* assigned reads could not be definitely identified as such. *Ralstonia solanacaerum* as obligatory and *C. acnes* as opportunistic human pathogens were assigned in all samples. The majority of the predominant bacteria species are putative contaminants of the human flora, but are also reported to cause severe infections, including *Haemophilus para-/influenza, Neisseria meningitides, Staphylococcus aureus*, or *pneumoniae*, or opportunistic infections, i.e., with *Burkholderia cepacia, Rothia mucilaginosa, Serratia marcescens*. Moreover, *Acinetobacter, Bacillus, Legionella, Mesorhizobium, Methylobacterium, Pseudomonas, Ralstonia*, and *Sphingomonas* genera amongst others have been related to contamination during sample preparation using different reagents (Salter et al., 2014). *Delftia acidovorans* and sp. Cs1-4, *Acinetobacter junii* and *baumannii, Rothia mucilaginosa*, and *Streptococcus thermophilus, mitis*, and *epidermidis* are in average the most represented bacteria species in the listed samples of patients (5.4, 2.0 (8.5), 3.8, 0.4 (4.8), 2.8 (3.2), 2.0, 1.4, 1.1 (4.5)%, respectively). These species along with the most of the other prominent bacteria were detected in the negative controls of the sample set as well as in negative controls of other institutions (Salter et al., 2014).

The parasites *Spirometra erinaceieuropaei as human obligatory pathogen* and the unexpected *Protopolystoma xenopodis* along with unexpected vertebrates other than human, *Apteryx australis mantelli, Cyprinus carpio, Mus musculus, Odocoileus virginianus texanus, Ovis canadensis canadensis, Sus scrofa*, and the plant *Triticum aestivum* were assigned in all the considered samples. Lastly, host NA depleted samples displayed generally more multifaceted profiles with more reads to the single species than the corresponding native preparation, except in the negative controls and the negative patCSF samples.

## Discussion

Metagenomics to screen for unclear or unknown infectious etiologies has been recently applied in several clinical cases to support routine diagnostics (Cordey et al., 2014; Naccache et al., 2015; Ortiz Alcántara et al., 2015; Kawada et al., 2016; Wylie et al., 2016; Lewandowska et al., 2017; Ruppé et al., 2017). Like for every eukaryotic sample, NGS analyses in clinical samples are greatly affected by a very low pathogen-to-host genome ratio. This challenge has been addressed in a variety of protocols for pathogen enrichment or host NA depletion (Allander et al., 2001; Hall et al., 2014; Jensen et al., 2015; Kohl et al., 2015; Lewandowska et al., 2015; Ruppé et al., 2017; Sabat et al., 2017). Nevertheless, these methods are focusing on a subset of pathogens, i.e., on bacteria or viruses (RNA or DNA). In this study, we developed a host NA depletion method that selectively lyses eukaryotic cells and preserves both, intact bacteria and viruses. Then the liberated eukaryotic NA are depleted and the remaining genomic material of bacteria and viruses is extracted. Subsequently, a reverse transcription and amplification is carried out on the extractives. Finally, a template preparation, the sequencing and data analysis are performed. The workflow was evaluated using a CSF surrogate of an infectious etiology and applied to samples of patients.

### Host NA depletion method effectively reduces host NA fraction and enables detection of low concentrations of pathogens

The proposed host NA depletion method achieved a net host NA reduction of 3100-fold with negligible net losses of Inf A virus and *Y.pseud*. bacterium in preliminary qPCR evaluations of surrogCSF replicates spiked with even pathogen concentrations. Three surrogCSF spiked with a 1:100 dilution series of Inf A virus confirmed stable results of host NA depletion. The net depletion of host NA was directly related to the method, while the rest was due to artifacts of normally lysed HUH7 cells and pathogens from cultures. For two samples spiked with Inf A virus dilutions (1:1, 1:100) four and two log cycles more reads, respectively, were assigned to the virus species due to host NA depletion. While no virus could be detected in the native 1:10,000 virus dilution, neither by qPCR nor by shotgun metagenomics, 34 reads (1.5E −4%) could be assigned to the spiked virus subtype following host NA depletion. The positive effect of the host NA depletion method on the spiked pathogens persisted also after the amplification processing (Supplementary Figure 7).

### Switch from WTA to two-step RT-WGA to include DNA viruses and support bacteria

Due to successful host DNA depletion and to prevent amplification favorable for host RNA the protocol was switched to two-step RT-WGA. Comparison to WTA revealed no notable difference regarding the recovery of spiked RNA virus, except for the percent reference coverage. Moreover, the overall sequencing quality specified by the number of usable raw reads after automatic quality filtering by the sequencing platform in relation to the loading of the sequencing chip resulted in a better overall sequencing quality for RT-WGA compared to WTA (ratio of 0.51 and 0.74, 0.33 and 0.16 for native and host NA depleted samples, respectively). Our approach composed of a separate RT followed by a WGA step supported our aim to combine bacteria and virus shotgun metagenomics workflow, thus enabling detection of DNA viruses (e.g., HSV-1; Whitley and Kimberlin, 2005) causing severe CNS infections. Besides, a second RT-WGA kit combination consisting of the SunScript™ Reverse Transcriptase RNase H- or H+ Kit and the TruePrime™ Single Cell WGA Kit (Version 1.0, Sygnis AG) using a primase instead of a random hexamer is a truly unbiased amplification without read shortening and contamination by adapter sequences. However, even after several optimizations coverage depth was poor. Although the overall sequencing quality was the best for the native samples, it was clearly not ideal for the host NA depleted samples (ratio of 0.68 and 0.08).

### Rapid and moderate comprehensive sequencing analysis followed by exhaustive remnants analysis

The proposed Kraken and BLAST® bioinformatics pipeline unifies a straightforward virus and bacteria taxonomic classification by Kraken and a slower confirmation part for the completion of recent or unexpected species by BLAST®. Initially, preliminary tests were performed to reduce data and increase the specificity by comparing several tools for a *de novo* assembly. Tools included MIRA (Chevreux et al., 1999, 2000), using the Overlap-Layout-Consensus assembly and a greedy approach algorithm, IDBA-UD (Peng et al., 2012), especially designed for uneven sequencing depths of metagenomic datasets, and SPAdes (Bankevich et al., 2012), which was already successfully applied to metagenomics analyses (Meiser et al., 2017; Papudeshi et al., 2017). None of these tools improved the analyses of our data neither focusing on the taxonomical classification nor the reference alignment (data not shown). Similarly, a clustering approach using CD-HIT (Li and Godzik, 2006; Fu et al., 2012) or UCLUST (Edgar, 2010) resulted in a vast number of clusters containing just a few reads. This was not surprising after identification e.g., of various contaminations which increased complexity of samples. Moreover, a pathogen screening approach performing alignments to a custom reference database using STAR (Dobin et al., 2013) or Bowtie2 (Langmead and Salzberg, 2012) was considerably more time consuming compared to Kraken. Finally, a taxonomical classification using Centrifuge (Kim et al., 2016) was promising to substitute Kraken/BLAST® analysis due to its ability to use the whole nt database requiring only processing power of a conventional desktop computer. Due to Centrifuge's algorithm to assign a read to up to 5 taxonomic categories, in contrast to Kraken/BLAST® assigning one read to one match, interpretation of metagenomic profiles prove to be more challenging. Moreover, Kraken was benchmarked to other tools and scored in the top range for speed, sensitivity and selectivity (Wood and Salzberg, 2014; Tausch et al., 2015; Kim et al., 2016; Lindgreen et al., 2016; Menzel et al., 2016). Kraken performs best, when all expected species of a sample are represented in the reference database (Wood and Salzberg, 2014). The quality of results of Kraken relies on an appropriate database. The custom database used in this study includes complete reference or representative genomes of all available viruses and bacteria as well as a human genome. Preliminary results emphasized the importance to include e.g., a human genome into the database, as otherwise a preeminent number of reads were false-classified to the human endogenous retroviruses. Adding to the issue, it was discovered, that representative reference genomes like carp and sweet potato, are contaminated by adapter sequences or bacterial DNA (http://www.opiniomics.org/we-need-to-stop-making-this-simple-fckingmistake/).

### A straightforward and rapid workflow suitable for clinical application

The developed host NA depletion method is straightforward and requires only 10 min hands-on and 50 min incubation time. The enzymatic and bead depletion steps can be performed using a pipetting robot. Adding automated NA extraction, 90 min machine run time follows. Reverse transcription and amplification of RNA along with DNA and following purification requires 30 min hands-on and 3.3 h machine run time. Ion Torrent™ library preparation, template preparation (o/n), sequencing and base-calling for one Ion 540™ Chip on an Ion S5™ XL take 18.6 h. Subsequent bioinformatics analysis up to assessment of the taxonomic profile of Kraken takes on average 1 h. With developing sequencing technique, the sequencing time will decrease promoting an even faster turnaround time (Greninger et al., 2015).

### Application to clinical samples

The effectiveness of the host NA depletion method to shift the NA ratio to the spiked pathogens in the surrogCSF could not be reproduced in host NA depleted CSF samples of patients. Three patCSF, previously defined by diagnostic qPCR to have approximately 1:100 dilution steps in pathogen concentration (Ct-value 24.1, 30.0, 35.5), were compared to the surrogCSF spiked with similar 1:100 dilution series of Inf A virus (Ct-value 20.7, 28.8, no qPCR detection). Reads of the previously diagnosed pathogens were assigned by Kraken/BLAST® analysis in each of the patCSF, but only in the native and none in the host NA depleted samples. Besides, the percent of assigned reads in the patCSF samples did not reflect the expected 1:100 pathogen dilutions compared to the surrogCSF, but resulted in dilutions of 1:16 and 1:4 (0.0034, 1.7E-4, 3.5E-5%, 283, 12, 3 reads, respectively). Accordingly, the nose swab presented fewer reads in the host NA depleted sample than in the native. This observation can presumably be ascribed to the delayed sampling in the convalescent phase. Importantly, the host NA depletion method was successfully applied in a serum of a patient with severe ANDV infection, where BLAST® analysis in the host NA depleted aliquot doubled the number of reads. This positive result of pathogen reads enrichment following host NA depletion is likely explained by the sample quality. After sampling, the serum was directly prepared for transport to the SPIEZ LABORATORY for diagnostic qPCR and NGS analysis. Another reason might be the integrity of the pathogen being less affected in serum than in CSF.

### Further metagenomic analysis in clinical samples

Except for the highest concentrated pathogen previously diagnosed by qPCR (VZV Ct-value 24.1, 0.0034%, 283 reads), no pathogen could be considered as obvious etiological cause in the remaining 12 patCSF analyzed by NGS (Table 1). Due to high background signal of the shotgun metagenomics profile, sorting the results for bacteria and viruses improved at least the evaluation of the viral results, preventing it from being overwhelmed by the dominating bacterial signals. Thereby, a suspected HSV-1 infection of a patCSF with Ct-value 30.0 (1.7E-4%, 12 reads, Table 1) could be confirmed in the native aliquot.

Additionally, Kraken/BLAST® results of previously detected pathogens by routine diagnostics were compared across a set of patCSF (5 viruses, 2 bacteria (Table 1 samples 10–23), and 1 negative). Kraken assigned a small number of reads to viral pathogens, but did not fulfill our prerequisites to be considered specific. Inconsistent with routine diagnostics, e.g., in the majority of samples a few reads were assigned to Herpes simplex virus 2 (HSV 2), a few to Varicella zoster virus in the negative CSF and no reads to JC virus. On this account, shotgun metagenomics profiles of clinical samples with low concentrations of pathogens have to be interpreted with caution. In general, metagenomics profiles should be interpreted in accordance with physicians' knowledge of underlying diseases. Despite, some publications base the detection or confirmation of a pathogen by shotgun metagenomics on just few reads, comparable to insignificant results due to high subsidiary pathogen signals of the present study (Feng et al., 2008; Palacios et al., 2008; Lewandowska et al., 2017).

The shotgun metagenomics bar graphs (Figure 5) of the most abundant species give insight into the complexity of shotgun metagenomics results. True species diversity of the sample and/or contaminations and noise are high and hardly distinguishable. A noise reduction can be achieved by ruling out species which have a low reads amount that are abundant across several species (Oechslin et al., 2017). The sensitivity of the shotgun metagenomics method is impressive as low concentrated RNA virus subtype spiked into surrogCSF could not be confirmed by qPCR. Bacterial and viral contaminations like *Ralstonia* spp. or *Legionella* spp. in NGS approaches observed in this study have already been identified to derive from commercial reagents, kit hardware like columns, and ultrapure water (Ralston et al., 1973; Kayser et al., 1975; Kulakov et al., 2002; van der Zee et al., 2002; Evans et al., 2003; Newsome et al., 2004; Peters et al., 2004; Adley et al., 2005; Shen et al., 2006; Naccache et al., 2013; Salter et al., 2014). Contamination by human flora during sampling has to be considered as well, but proves challenging as opportunistic CNS infections by *Rothia mucilaginosa, Comamonas testosteroni*, or *Delftia acidovorans* have been described (Ruoff, 2002; Arda et al., 2003; Jin et al., 2008; Lee Md et al., 2008; Yamane et al., 2010; Kaasch et al., 2011; Khan et al., 2012; Orsini et al., 2014; Ramanan et al., 2014; Bilgin et al., 2015; Salzberg et al., 2016).

While the detection of usually low concentrated viral and bacterial genetic material in clinical samples is impeded by the predominant host proportion, low concentrated samples (host NA depleted) have been reported to be especially susceptible to contamination and artifacts similar to overwhelming host proportions in native samples (Lusk, 2014; Perlejewski et al., 2015; Bukowska-Ośko et al., 2017). Furthermore, the unexpected detection of the M.c.c.a.V in surrogCSF and patCSF might be an artifact due to bioinformatics algorithms or the databases used, as M.c.c.a.V was also reported by Motooka et al. (2015), and may also be the cause of other unexpected species hits (Cantalupo et al., 2011; Laurence et al., 2014; Wood and Salzberg, 2014).

A similar study was performed by Hasan et al. (2016), where instead of surrogCSF, human CSF and nasopharyngeal aspirate samples were spiked with DNA viruses concentrations of Ct-values 21.0 to 24.6 followed by depletion using a detergent based selective host cell lysis and DNase treatment. A clinical nasopharyngeal aspirate with Human adenovirus of Ct-value 21.9 was analyzed to evaluate the procedure, which successfully enriched pathogen's read counts. On the contrary, human samples used in our study had Ct-values of 27 and higher (except sample 1, 2 Table 1 with Ct-value 24). Moreover, in contrast to our study, the CSF samples in Hasan's study have not been frozen before NGS analysis, which possibly affected the pathogens' integrity in our samples (Ruppé et al., 2017) in addition to be damaged e.g., due to advanced host immune response at the time of sampling or rapid administration of unspecific medication as an immediate measure. Unknown influences affecting shotgun metagenomics results might be revealed by reproducing Hasan's approach by spiking our clinical inflammatory CSF samples with defined pathogen concentrations. A comparison of our bead-beating to Hasan's detergent host cell lysis is of further interest.

In conclusion, the proposed host NA depletion method for shotgun metagenomics analysis successfully increased the reads proportion of intact viral and bacterial pathogens in spiked surrogCSF. Due to the sensitivity and non-selectivity of the whole shotgun metagenomics workflow, a high number of reads was assigned to the host portion and various human commensal bacteria and laboratory environment contaminants. Thus, metagenomics analyses do currently not permit the proposition of a clinically significant detection threshold. As routine diagnostics in patCSF usually deals with low concentrations of the pathogens, previous medication using antibiotics and antivirals as well as the sampling time point additionally impact the integrity and presence of viruses and bacteria in CSF.

Summarizing, shotgun metagenomics is a promising tool for supporting diagnostics of CNS infections, but is currently not applicable as a standalone method. The significance of the outcome is highly depending on sample quality, contamination by the human flora and laboratory environment, thus a proper sample preparation in addition to a well-curated and comprehensive reference database has to be implemented for future clinical use of shotgun metagenomics.

## Data availability statement

The sequencing datasets for this manuscript are not publicly available due to restrictions from the Ethical Committee (Ethics Commission of the Canton of Bern). Requests to access the datasets should be directed to Dr. Christian M. Beuret, christian.beuret@babs.admin.ch.

## Author contributions

NLe performed BSL-3 sample preparation of the serum until inactivation and revised the manuscript. NLi supported bioinformatics analysis and revised the manuscript. SR undertook four sequencing preparations and runs. PA provided samples and conducted the ethics committee application. RB contributed to interpretation of bioinformatics data. SL initiated and supervised the project academically. CB co-initiated the project with SL, coordinated the project, supported data analysis, and revised the manuscript. CO conducted the experiments, performed the in-house sequencings (exception mentioned), the bioinformatics analysis, the general data analysis, and wrote the manuscript. All authors read and approved the final manuscript.

### Conflict of interest statement

The authors declare that the research was conducted in the absence of any commercial or financial relationships that could be construed as a potential conflict of interest.

## Abbreviations

CNS: central nervous system
(q)PCR: (quantitative real-time) polymerase chain reaction
NA: nucleic acid(s)
CSF: cerebrospinal fluid
surrogCSF: surrogate sample for CSF of a central nervous system infection
patCSF: CSF sample of a patient
Inf A: Influenza A virus
*Y.pseud*.: Yersinia pseudotuberculosis
RT: reverse transcription
WGA: whole genome amplification
WTA: whole transcriptome amplification
nt: nucleotide database of the NCBI
Ct-value: cycle threshold value
gDNA: genomic DNA
mtDNA: mitochondrial DNA
rRNA: ribosomal RNA
stddev: standard deviation.
