# Supplementary material for: Limited Correlation of Shotgun Metagenomics Following Host Depletion and Routine Diagnostics for Viruses and Bacteria in Low Concentrated Surrogate and Clinical Samples
Source: Front Cell Infect Microbiol. 2018 Oct 23;8:375. doi: 10.3389/fcimb.2018.00375 (PMC6206298; doi:10.3389/fcimb.2018.00375)
Supplement: Supplementary file 1 [file Data_Sheet_1.docx]

Supplementary Material

Limited Correlation of Shotgun Metagenomics Following Host Depletion and Routine Diagnostics for Viruses and Bacteria in Low Concentrated Surrogate and Clinical Samples

Corinne P. Oechslin, Nicole Lenz, Nicole Liechti, Sarah Ryter, Philipp Agyeman, Rémy Bruggmann, Stephen L. Leib, Christian M. Beuret^*^

*** Correspondence:** Dr. Christian M. Beuret: Christian.Beuret@babs.admin.ch

# Supplementary Data

## Materials and Methods

### Surrogate CSF samples (surrogCSF)

Immortalized cell lines were used to model a surrogate inflammatory CSF of a CNS infection. Initially, A9 mouse hybridoma B lymphocyte cells provided by Prof. Dr. Christoph Kempf at the Zentrallaboratorium Bern (ZLB) were used for method evaluation of the selective host cell lysis. In regard to evaluation of the bioinformatics analysis workflow for later application to clinical human samples, A9 cells were substituted by human cells (human hepato-cellular carcinoma cells, HUH7). HUH7 cells present a comparable susceptibility to bead‑beating lysis (data not shown) and were used for depletion of free floating NA up to bioinformatics method development.

### Pathogens

SurrogCSF were spiked first using *Streptococcus pneumoniae* (3908.41, Institute for Infectious Diseases, University of Bern) (o/n at 37°C on CO_2_ preconditioned blood agar plate) as bacterial model. *S. pneumoniae* are Gram-positive bacteria being potentially more stable regarding mechanical stress due to their thicker cell wall and their polysaccharide capsule than Gram-negative bacteria. Hence, the bacteria model was switched to Gram-negative *Escherichia coli* (ATCC 25922, Oxoid AG) (o/n at 37°C on tryptic soy agar plate) and finally to *Y. pseudotuberculosis* (*Y.pseud.*) for assessing bacteria loss during the host NA depletion method. Besides the (-)ssRNA Influenza A H3N2 virus (Inf A) used as viral model, for a NA extraction kit comparison Zika virus was used (enveloped, (+)ssRNA) cultures (Zika virus/Uganda 976/NCPV MP1751, VERO E6).

### Development of the host NA depletion method

The selective host cell lysis and the depletion of the thereby released free-floating NA were developed in parallel. During development, the method was first optimized for RNA viruses followed by bacteria tests.

#### Selective host cell lysis

Previous to the final method using a bead‑beater Precellys™ 24 tissue homogenizer with a Soft tissue homogenizing Lysing Kit, preliminary tests were performed. Initially, repeated freezing (‑20°C) and thawing (4°C) for 1 to 3 times was tested (data not shown). Osmotic lysis was tested by adding 0.7X sample volume 18 MΩ.cm water (Milli-Q® Direct Water Purification System, Merck KGaA). Then, combinations were tested of two freeze/thaw cycles and osmotic lysis with subsequent ultrasonication as well as the combination of ultrasonication and bead‑beating lysis using the Precellys™. Therefor, samples were brought to 100 µl by adding 18 MΩ.cm water, vortexed for 15 seconds, centrifuged for 10 seconds, placed on ice for 15 minutes and then sonicated by either the Bioruptor® Standard sonication system (Diagenode Inc.) or the Covaris® M220 Focused-ultrasonicator™ (Covaris, Inc.). The Bioruptor® settings were chosen to lyse host cells while leaving bacteria intact: Power: Low, Sonication cycle: 30 seconds ON/30 seconds OFF, Temperature: 4°C, Total sonication time: 4 cycles (compare to the “Bacterial Cell Disruption” and “RNA extraction from tissue” Bioruptor® protocols). The Covaris® was used with a microTUBE AFA Fiber Snap‑Cap tube and the run protocol for a DNA shearing target peak of 1000 bp was followed according to the manual: Peak Incident Power 50 W, Duty Factor 5%, Cycles per Burst 200, Treatment Time 60 seconds, Temperature 20°C. The lysis efficiency of all methods was evaluated by performing depletion of free-floating NA using AMPure® XP paramagnetic beads reagent and subsequent qPCR detection.

#### Depletion of free-floating NA

Several methods were evaluated before adding a Benzonase® nuclease step to the host NA depletion protocol in order to degrade free‑floating NA before depletion by AMPure® XP paramagnetic beads reagent. An initially tested AMPure® XP based capture of the released NA without previous nuclease treatment was improved by performing twice the NA capture, before and after the lysis step. To overcome the resulting high sample volume (2 ml) for the subsequent automated NA extraction, bead suspensions of altered component concentrations were tested. A buffer similar to the commercial AMPure® XP reagent composed of 20% polyethylene glycol (PEG), 2.5 M NaCl and water was used [271]. Thereby, the AMPure® XP beads were immobilized on a magnet and the original buffer was removed. Then, the beads in different amounts were suspended in different PEG‑NaCl buffers, *e.g.* 1X beads in 0.33X volume of 3X PEG 0.6X NaCl, and applied either once after or twice before and after beat‑beating lysis using a Precellys™.

Furthermore, the efficiency of NA depletion of AMPure® XP beads was compared to the one of TurboBeads® (Turbobeads LLc.). AMPure® XP beads are micro‑sized polysterene beads with an outer layer of magnetite and coated by negatively charged carboxyl groups (m‑beads) suspended in a crowding agent buffer. The TurboBeads® are nano‑sized metal core beads with a thin layer of graphene‑like carbon and different coatings, suspended in water. TurboBeads® PEG‑Carboxy (outer layer PEG with COO^-^ coating, 30 mg/ml, Cat No.: 4003) (n‑beads) were sonicated before usage with a Bioruptor® Standard at high power for 5 minutes. Then 0.5X sample volume beads were immobilized on a magnet, the water was removed and the beads were suspended in 1X PEG‑NaCl buffer. The TurboBeads® PEG‑Carboxy were then tested in place of AMPure® XP beads with a previous Benzonase® nuclease treatment or by adding the bead suspension to the sample at the bead‑beating lysis and proceeded afterwards at the point of paramagnetic beads incubation without nuclease step.

Moreover, the application of antibody (Ab) coated paramagnetic beads was tested too. Micro‑sized paramagnetic beads (Dynabeads® M-270 Epoxy) were coated with mouse anti-human nuclei (clone 235-1, MAB1281, Merck KGaA) and mouse anti-human mitochondria (clone 113-1, MAB1273, Merck KGaA) monoclonal antibodies using the Dynabeads™ Antibody Coupling Kit (Invitrogen™, Thermo Fisher Scientific Inc.) according to manufacturer’s protocol (10 µg Ab per mg of beads; 0.05% Tween added to LB and HB buffer; elution in SB to final concentration of 10 mg/ml). Incubation for Ab coupling was done using a Multi Bio RS‑24 (Biosan) (settings: orbital: 14 rpm, 5 seconds; reciproc: 45°, 20 seconds; vibro: 5°, 5 seconds; 24 hours) placed in an incubator at 37°C. Two micro liters of the human nuclei and 6.8 µl of the mitochondria Ab coated beads (Ab‑beads) were added to a sample after the AMPure® XP step, once after or twice before and after beat‑beating lysis. Incubation was performed at 4°C for 1 hour on a Multi Bio RS-24 (settings as for Ab coupling respectively).

#### NA extraction

Two automated and three manual NA extraction methods were compared and methods were performed according to the manufacturer’s protocol. Samples extracted using an automated system were previously inactivated for biosecurity reasons by adding 4X volume AVL buffer (Qiagen N.V.), vortexed for 10 seconds and incubated at room temperature for 10 minutes. The automated systems used were the MagNA Pure 96 system with the DNA and Viral NA Large Volume Kit and Viral NA Universal LV 1000 3.0.1 run protocol (500 and 1000 µl input, 50 and 100 µl elution volume) (Roche Diagnostics International AG) (applied method if nothing else is stated), and the EZ1 Advanced system with the EZ1 Virus Mini Kit v2.0 (400 µl input, 60 µl elution volume) (Qiagen N.V.). The manual methods included the RNeasy Plus Universal Mini Kit (Qiagen N.V.), the QIAamp Viral RNA Mini Kit (Qiagen N.V.) and the High Pure Viral RNA Kit (Roche Diagnostics International AG). An additional altered RNeasy Kit protocol was performed to allow total NA extraction. Therefore, the gDNA Eliminator Solution of the original protocol was skipped and the pH of the sample‑QIAzol mixture was raised to pH of approximately 8 by adding 10 M NaOH (EMPROVE® bio, Merck KGaA) in a ratio of 1/28X volume.

### qPCR analysis

For the development of the host NA depletion method, different reagents and methods were evaluated and results were compared using qPCR analysis. The primer systems additionally used were TaqMan® Gene Expression Assay ACTB mouse (Mm00607939_s1) for A9 cells, an AquaScreen® Escherichia coli qPCR Detection Kit (Minerva Biolabs® GmbH) for *E. coli*, and the validated in‑house primer‑probe set lytA for *S. pneumoniae*.

## Results

### Selective lysis of host cells

Several methods to selectively lyse A9 host cells were compared: osmotic lysis, bead‑beating, focused high frequency (HF) and bath low frequency (LF) ultrasonication, the latter in combination with preceding repeated freezing (‑20°C) and thawing, and a beat‑beating step. The lysis efficiencies for native and host NA depleted surrogCSF spiked with Inf A and partly with either *E. coli* or *S. pneumoniae* (Supplementary Figure 2) were compared using qPCR. The bead-beating host cell lysis methods exceled all other tested methods in releasing host gDNA copies at least by a factor 10.5, whereas the loss of Inf A RNA copies differed merely within a factor 10 between all the methods.

### Depletion of free-floating NA

Several methods to deplete free‑floating NA in surrogCSF after beat‑beating lysis of host cells were evaluated by qPCR targeting host gDNA, mtDNA and rRNA and spiked bacteria and viruses. To assess the net amount of depleted NA after host cell lysis, naturally free‑floating NA in surrogCSF were previously depleted using AMPure® XP paramagnetic beads reagent (COO^-^ coated micro‑paramagnetic beads (m‑beads)). Then, the efficacies of depletion of TurboBeads® PEG‑Carboxy (PEG-COO^-^ coated paramagnetic nano‑beads (n-beads)) and COO^‑^ or Ab coated micro‑paramagnetic beads (m-beads, Ab‑beads) were compared using different method settings. The more, the efficacies of n- and m-beads were assessed by implementing a preliminary Benzonase® nuclease treatment to improve the depletion of large host NA. Summarized, in Supplementary Figure 3 the following six method combinations were compared:

1. Adding n-beads to the host cell lysis step (n‑beads, while host cell lysis)
2. Performing two NA depletions using m-beads followed by an additional Ab‑beads mediated organelles depletion, before and after (pre+post) the host cell lysis step (Ab‑beads following m‑beads, pre+post)
3. Performing two NA depletions by m-beads, pre+post host cell lysis step (m‑beads pre+post)
4. A depletion using a concentrated m-bead buffer (1X beads in 0.33X volume of 3X PEG 0.6X NaCl) pre+post host cell lysis step (concentrated m‑beads pre+post)
5. A nuclease treatment prior to a NA depletion using n-beads after (post) the host cell lysis step (nuclease, n‑beads, post)
6. A nuclease treatment prior to a NA depletion using m-beads post host cell lysis step (nuclease, m‑beads, post)

A nuclease treatment prior to a NA depletion using m-beads presented the highest net depletion of host gDNA (ΔCt-value 11.4±1; factor 2630±2 standard deviation (stddev), 5 replicates) and the lowest net losses of virus and bacterium (ΔCt-value 0.3±0.9; factor 1.2±1.8 stddev, 5 replicates, and ΔCt-value 0±0.9; factor 1±1.8 stddev, 4 replicates). The method using n‑beads (method 5) yielded the second highest net depletion of host gDNA (ΔCt-value 9.8±1.8; factor 841±3.3 stddev, 2 replicates) and the highest net depletion of host rRNA (ΔCt-value 12±3; factor 3981±7.9 stddev, 2 replicates), but also a considerable loss of viruses compared to m‑beads (ΔCt-value 2.5±1.5; factor 5.6±2.8 stddev, 2 replicates). Methods 1–4 omitting a prior nuclease treatment performed worse at the subsequent depletion. Thereby, the use of a concentrated m-bead buffer showed the highest net depletion for host NA, especially for mtDNA, but also a remarkable depletion of viral and bacterial NA. Performing a host NA depletion using the m‑beads twice, before and after the lysis step (method 3), even with an additional Ab-beads step for host nuclei and mitochondria (method 2), did not improve the host NA depletion.

### NA extraction

#### RNA viruses

RNA virus detection in clinical samples by metagenomics is very critical due to the small genome sizes and the overall instability of RNA. Therefore, five NA extraction methods were compared for subsequent Ion Torrent™ sequencing. The input for each kit was 100 µl of Zika virus culture supernatant (ZIKV) and bioinformatics analysis using Kraken, BLAST® and Bowtie2 were performed. The hands‑on RNeasy Plus Universal Mini Kit (Qiagen N.V.) yielded the highest number of reads assigned to ZIKV (15.6% of total reads) after filtering and trimming raw reads. The automated paramagnetic bead system MagNA Pure 96 (DNA and Viral NA Large Volume Kit, Viral NA Universal LV 1000 3.0.1), the hands‑on High Pure Viral RNA Kit (Roche Diagnostics International AG) and the QIAamp Viral RNA Mini Kit (Qiagen N.V.) yielded similar results of 4.4, 3.9, and 3.5%, respectively. The EZ1 automated paramagnetic bead system (EZ1 Virus Mini Kit v2.0) disclosed the lowest number of ZIKV specific reads (1.8% of total reads) (Supplementary Figure 4 A). Likewise, the reference alignment of ZIKV showed the highest reference coverage of 99.7% (1.7% maximum depth of coverage) by the RNeasy Plus Universal Mini Kit with 14.9% of total reads aligned. The MagNA Pure 96 DNA with Viral NA Large Volume Kit, the High Pure Viral RNA Kit, the QIAamp Viral RNA Mini Kit and the EZ1 Virus Mini Kit v2.0 yielded lower and similar values of 99.1, 98.8, 99.0, and 98.9% reference coverage (0.4, 0.5, 0.4, 0.2% maximum depth of coverage) and 4.2, 3.7, 3.4, and 1.7% of total reads aligned (Supplementary Figure 4 B).

#### Inclusion of DNA viruses and bacteria

The RNeasy Plus Universal Mini Kit (Qiagen N.V.) was adapted to include the extraction of viral and bacterial DNA. Briefly, the pH of the guanidinium‑thiocyanate-phenol‑chloroform (GT-PC) reagent (QIAzol, Qiagen N.V.) was increased to pH 8 by adding NaOH. The extracted NA was subsequently purified using the automated MagNA Pure 96 (Roche Diagnostics International AG) system. The pH adapted GT‑PCpH8 extraction method was compared to the performance of the MagNA Pure 96 system using the guanidinium‑thiocyanate (GT) based DNA and Viral NA Large Volume Kit and Viral NA Universal LV 1000 3.0.1 run protocol for native and host NA depleted surrogCSF. The qPCR results demonstrated the efficacy of host NA depletion method. There was a decrease in the copy number of human gDNA, mtDNA and rRNA (5.5, 2, 4 log cycles, respectively) and depicted a minor loss of spiked and already released viral and bacterial NA (1.5, 2 log cycles, respectively). Additionally, the qPCR comparison of the GT to the GT‑PCpH8 based NA extraction revealed no difference for native and host NA depleted surrogCSF (Supplementary Figure 5). The comparison of the Ion Torrent™ sequencing results for host NA depleted samples presented an increase in the number of assigned reads for spiked pathogens extracted using the GT compared to extraction by GT‑PCpH8 method (GT: factor 640 Inf A and 3 *Y. pseud.*, GT‑PCpH8: 35 Inf A and 2 *Y. pseud.*) (Supplementary Figure 6 A). Further considering classified pathogen reads, the host NA depletion combined with GT method was more effective than host NA depletion combined with GT‑PCpH8 method. It generated more RNA virus (0.4, 0.08% total reads after filtering and trimming), but less bacterium reads (27, 35%) by Kraken/BLAST®. Likewise, the native virus spiked surrogCSF yielded more (6.2E-4, 2.1E-3%) and the native bacteria spiked sample less (9, 17%) reads using the GT compared to the GT‑PCpH8 extraction method (Supplementary Figure 6 A). The reference alignment by Bowtie2 supported overall the RNA virus and bacteria results of the taxonomical classification by Kraken/BLAST® (Supplementary Figure 6 B, C). Nonetheless, the Bowtie2 results of the bacteria spiked surrogCSF showed in general smaller alterations between the different preparations than the virus spiked samples. The % reference coverage of *Y. pseud.* was equally high or lower while the maximum depth of coverage was higher for the GT method than that of the GT‑PCpH8 method (native: 24 and 0.1, resp. 40 and 0.05%, host NA depleted: 90 and 0.1, resp. 91 and 3%).

The bacteria spiked surrogCSF presented high bacteria concentrations. They displayed a less effective enrichment by the host NA depletion than the virus spiked surrogCSF showing a lower pathogen concentration. Though, in the simultaneously prepared virus spiked surrogCSF the bacteria contamination signal pictured a similar enrichment compared to the virus signal. Accordingly, while in the native sample the viral and bacterial signal denoted 4 and 0 reads after host NA depletion 3,100 and 5,100 reads were detected, respectively.

# Supplementary Figures and Tables

## Supplementary Figures

**(A)
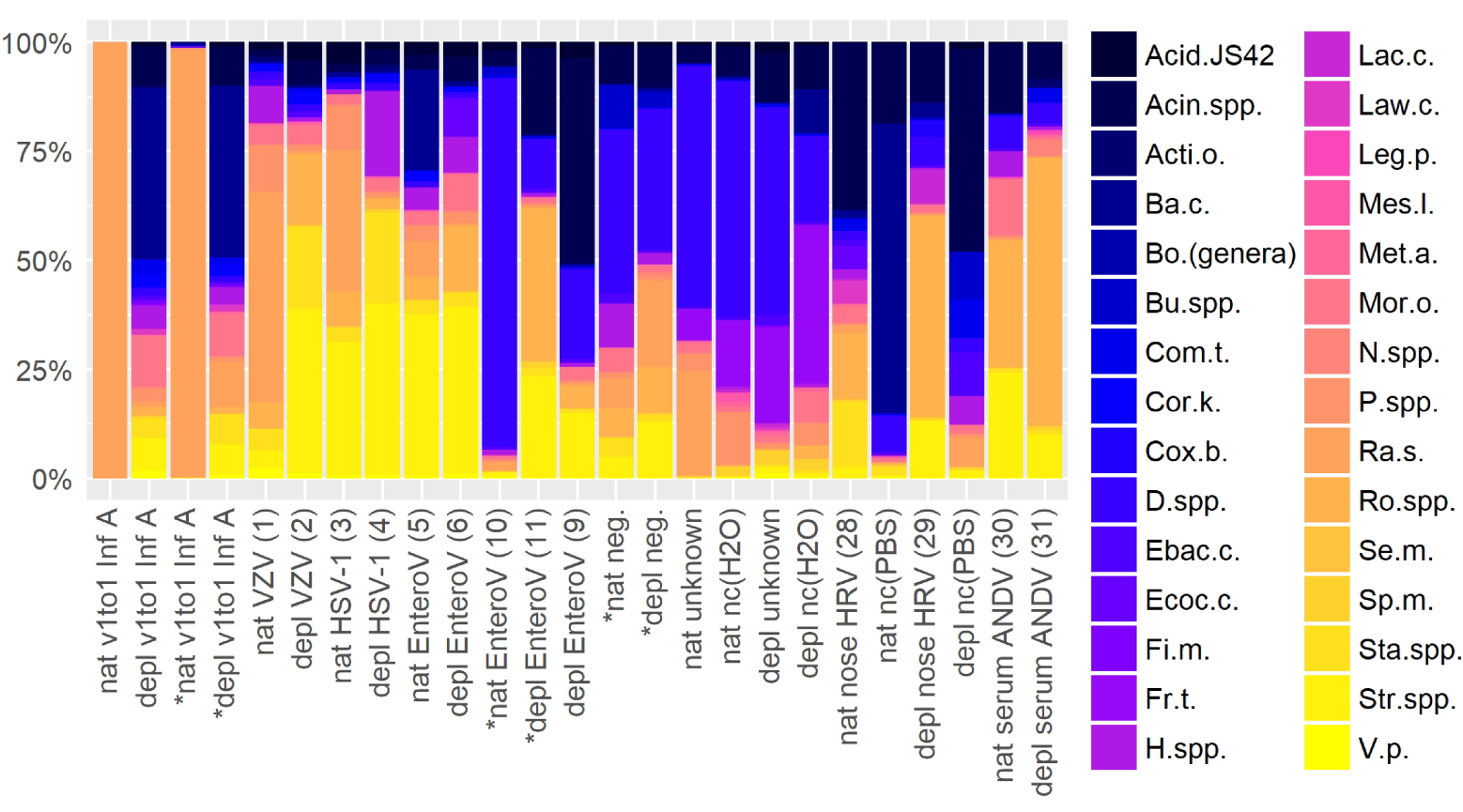
**

**(B)**
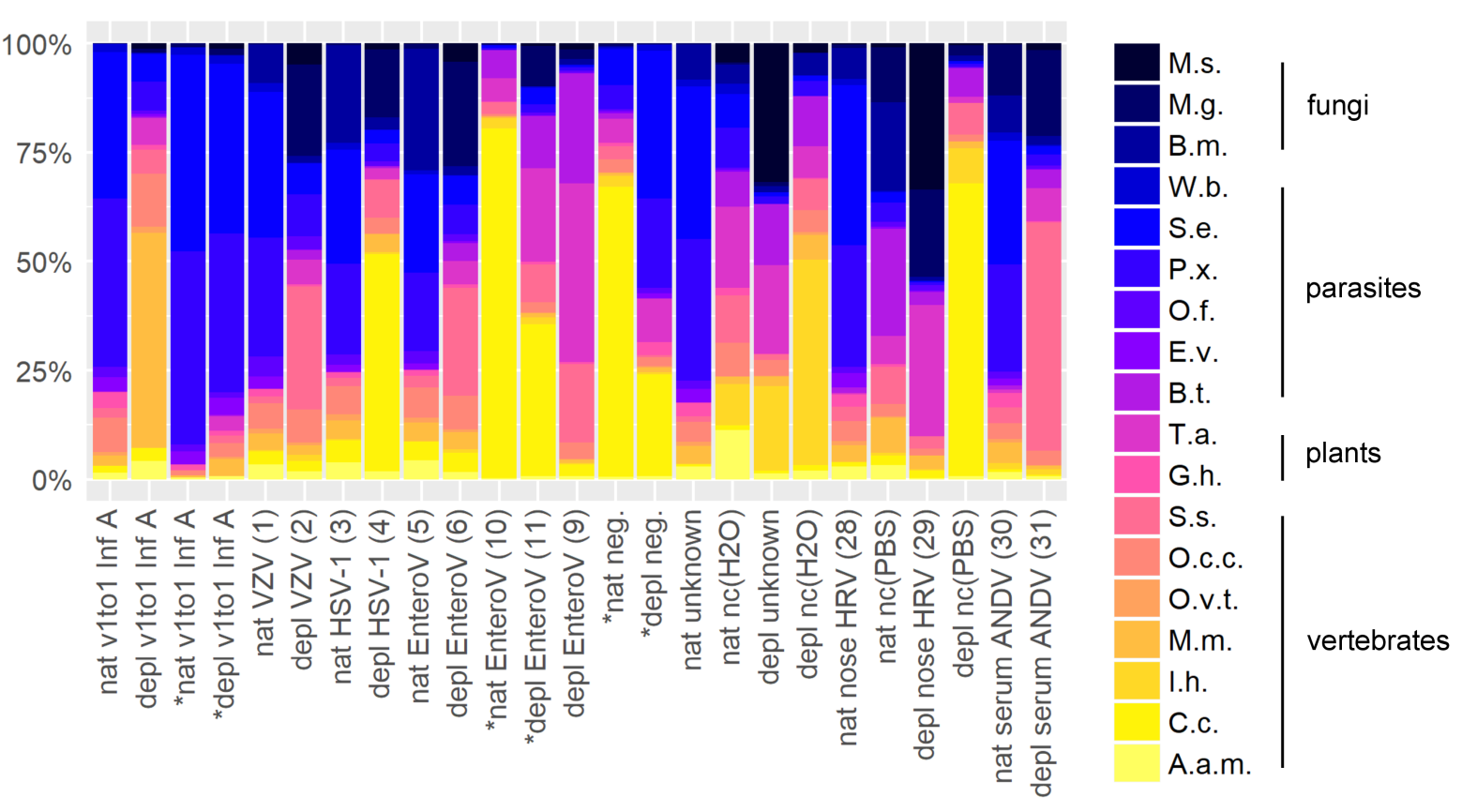


**Supplementary Figure 1.** **Abundance overview of most prominent bacteria (A) and eukaryote (B) species in metagenomics profiles in at least one across a set of different samples** comprising native (nat) and host NA depleted (depl) surrogate CSF samples spiked with Influenza A virus (Inf A) and amplified using whole transcriptome amplification (WTA2) and samples of patients and negative controls (nc) amplified using sequential reverse transcription and whole genome amplification (SuperScript™ III First‑Strand Synthesis SuperMix, PicoPLEX® WGA Kit) including CSF samples of patients the pathogen was detected in high (VZV) and low (HSV‑1) amounts, diagnostically negative (neg.), and of unknown disease etiology accompanied by PCR grade water nc (H2O), furthermore a nose swab (nose HRV) accompanied by PBS nc, and a serum (serum ANDV). Majority of samples were sequenced on an Ion Torrent™ Ion S5™ using Ion 540™ Chips, few were sequenced on an Illumina® HiSeq 2500 V4 paired‑end (*) on ^1^/_2_ lane (nat and depl EnteroV (samples 10, 11 of Tab.1), nat and depl neg.), respectively on ^1^/_6_ lane (nat and depl v1to1 Inf A). Abbreviations: VZV =Varicella zoster virus, HSV‑1 =Herpes simplex virus 1, EnteroV =Enterovirus genus, HRV =Human rhinovirus, ANDV =Andes virus, **A:**Acid.JS42 =*Acidovorax* sp. *JS42*, Acin.spp. =*Acinetobacter* spp., Acti.o. =*Actinomyces oris*, Ba.c. =*Bacillus cereus*, Bo.(genera) =*Borrelia/-ella*, Bu.spp. =*Burkholderia* spp., Com.t. =*Comamonas testosteroni*, Cor.k. =*Corynebacterium kroppenstedtii*, Cox.b. =*Coxiella burnetii*, D.spp. =*Delftia*spp., Ebac.c. =*Enterobacter cloacae*, Ecoc.c. =*Enterococcus cecorum*, Fi.m. =*Finegoldia magna*, Fr.t. =*Francisella tularensis*, H.spp. =*Haemophilus* spp., Lac.c. =*Lactobacillus crispatus*, Law.c. =*Lawsonella clevelandensis*, Leg.p. =*Legionella pneumophila*, Mes.l. =*Mesorhizobium loti*, Met.a. =*Methylobacterium aquaticum*, Mor.o. =*Moraxella osloensis*, N.spp. =*Neisseria spp.*, P.spp. =*Pseudomonas spp.*, Ra.s. =*Ralstonia solanacearum*, Ro.spp. =*Rothia* spp., Se.m. =*Serratia marcescens*, Sp.m. =*Sphingomonas melonis/*sp. *TY*, Sta.spp. =*Staphylococcus* spp., Str.spp. =*Streptococcus* spp., V.p. =*Variovorax paradoxus*, **B:**A.a.m. =*Apteryx australis mantelli*, C.c. =*Cyprinus carpio*, I.h. =*Isichthys henryi*, M.m. =*Mus musculus*, O.v.t. =*Odocoileus virginianus texanus*, O.c.c. =*Ovis canadensis canadensis*, S.s. =*Sus scrofa*, G.h. =*Gossypium hirsutum*, T.a. =*Triticum aestivum*, B.t. =*Brugia timori*, E.v. =*Enterobius vermicularis*, O.f. =*Onchocerca flexuosa*, P.x. =*Protopolystoma xenopodis*, S.e. =*Spirometra erinaceieuropaei*, W.b. =*Wuchereria bancrofti*, B.m. =*Bipolaris maydis* ATCC 48331, M.g. =*Malassezia globosa*, M.s. =*Malassezia sympodialis*.

**
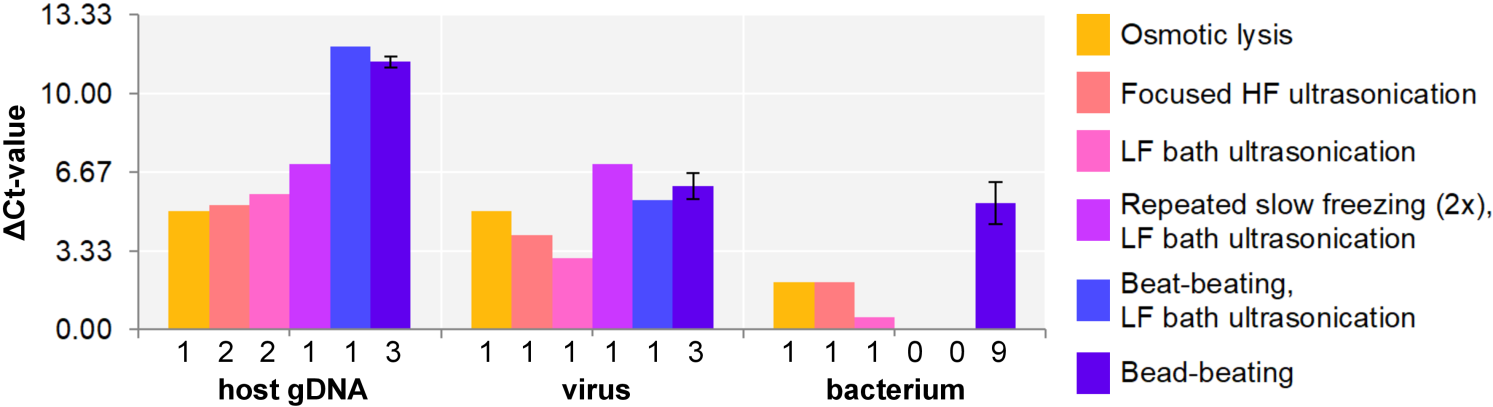
**

**Supplementary Figure 2. Six selective host cell lysis methods evaluated by qPCR.** Primer-probe targets listed on x-axis: the actin B gene host cell genomic DNA (gDNA), the matrix proteins of Influenza A virus (virus), the transcriptional regulator uidR of *E. coli* (beat-beating method) and the pneumococcal autolysin virulence factor lytA of *S. pneumoniae* (osmotic lysis, focused HF and LF bath ultrasonication) (bacterium). The cycle threshold value (ΔCt-value) describes the total amount of free NA that were depleted from the suspension after the particular lysis method of a host NA depleted compared to a native surrogate CSF sample. The y-axis shows ΔCt-value changes of 3.33 per steps, representing an approximated tenfold reduction of copy number. The number of performed tests is indicated on the bottom of each bar. The error bars depict the standard deviation of the mean Ct-value of replicates in number >2.

**(A)**

**
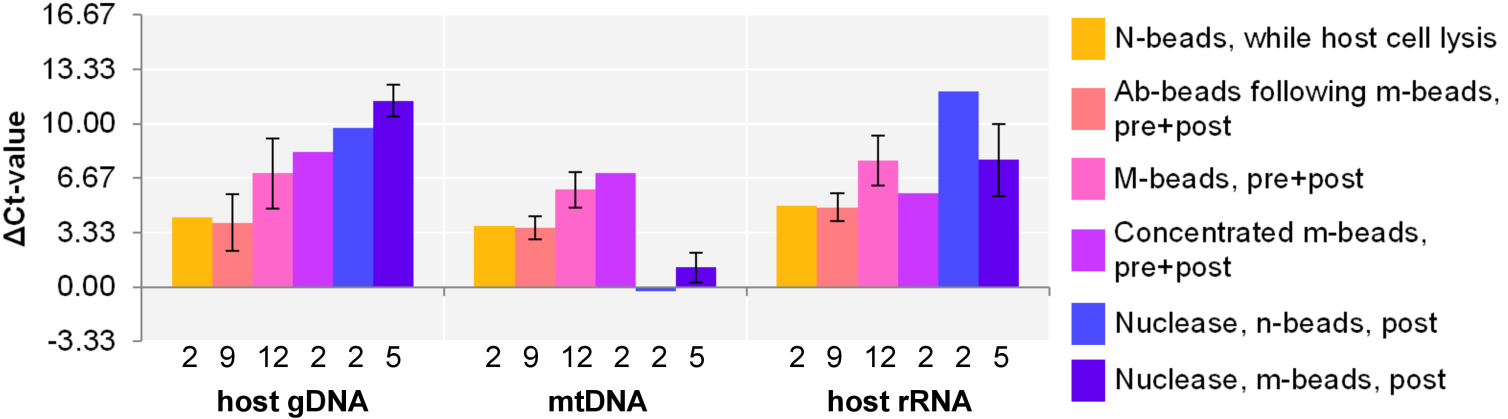
**

**(B)**

**
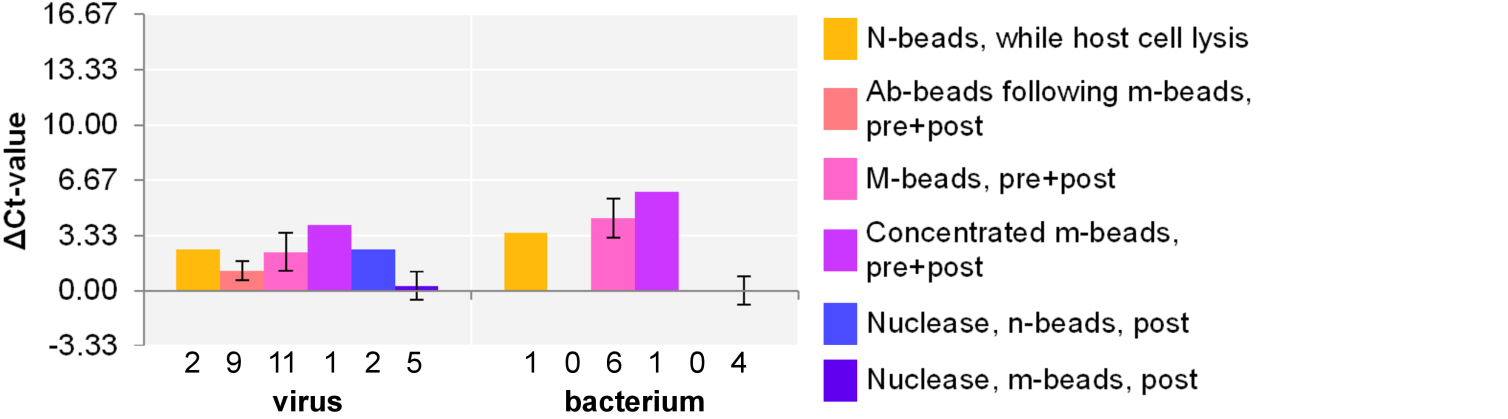
**

**Supplementary Figure 3.** **Six free‑NA depletion methods after beat‑beating host cell lysis compared by qPCR**. Primer-probe targets listed on x‑axis: **A:** the actin B gene host cell genomic DNA (gDNA), the mitochondrial NADH-ubiquinone oxidoreductase chain 1 DNA (mtDNA) and the eukaryotic 18S ribosomal RNA (host rRNA), **B:** the matrix proteins of Influenza A virus (virus), the putative siderophore biosynthesis protein of *Y. pseudotuberculosis* (n‑beads while host cell lysis, m‑beads pre+post and with previous nuclease step methods) and the transcriptional regulator uidR of *E. coli* (m‑beads pre+post and concentrated beads suspension methods) (bacterium). The cycle threshold value (ΔCt-value) describes the net amount of depleted NA by each depletion method after host cell lysis of a host NA depleted compared to a native surrogate CSF sample. The y‑axis shows ΔCt-value changes of 3.33 per steps, representing an approximated tenfold reduction of copy numbers. The number of performed tests is indicated on the bottom of each bar. The error bars depict the standard deviation of the mean ΔCt-value of the technical replicates in number >2. Abbreviations: n‑beads =nano‑sized paramagnetic beads (TurboBeads® PEG‑Carboxy); while host cell lysis =n‑beads were added to the beat‑beating host cell lysis step; Ab‑beads =antibody coated micro‑sized paramagnetic beads (anti-human nuclei, anti‑human mitochondria), m‑beads =micro‑sized paramagnetic beads (AMPure® XP); pre =previous to host cell lysis; post =following host cell lysis; concentrated =1X m‑beads in 0.33X volume of 3X PEG 0.6X NaCl (DeAngelis et al., 1995); nuclease =Benzonase® nuclease treatment previous to paramagnetic beads application.

**(B)**

**(A)**

**
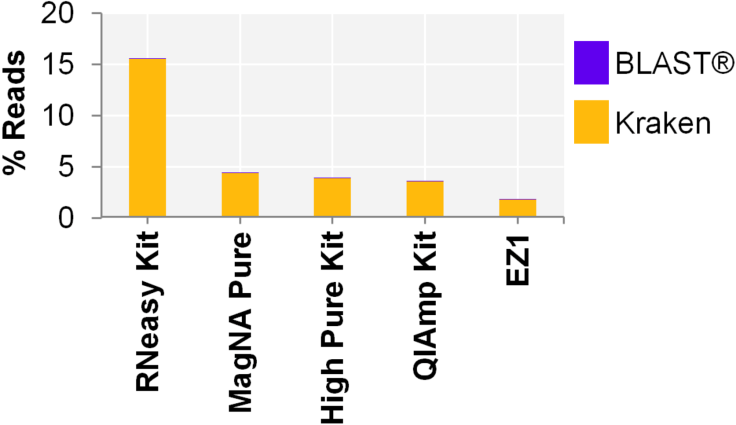

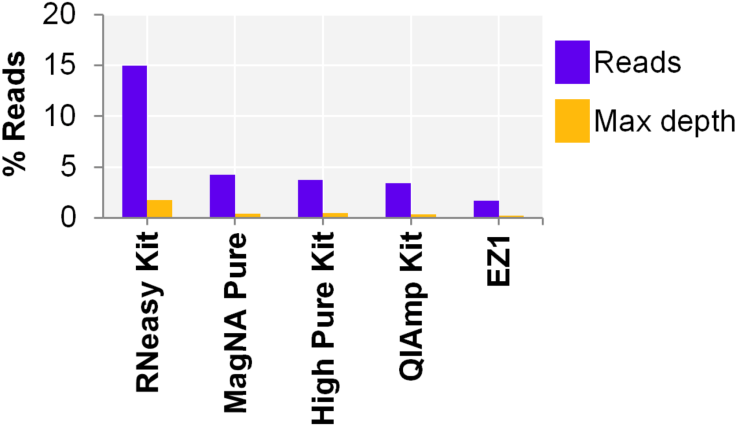
**

**Supplementary Figure 4.** **Efficacies of five RNA extraction methods for subsequent NGS analysis** by comparing viral RNA of Zika virus (ZIKV) hits of **A:** taxonomical classification of reads by exact k‑mer mapping (Kraken) to a custom database of human, viruses and bacteria assemblies from RefSeq or Genbank® complemented with local alignment to the NCBI’s nt (BLAST®) of unclassified reads by Kraken and **B:** reference alignment to ZIKV (Bowtie2) with number aligned reads and maximum depth of coverage (Max depth). X‑axis indicates the NA extraction methods and the y‑axis shows the number reads in % respective to the total reads that were taxonomically classified or assigned as unclassified by Kraken in decimal logarithm scale.


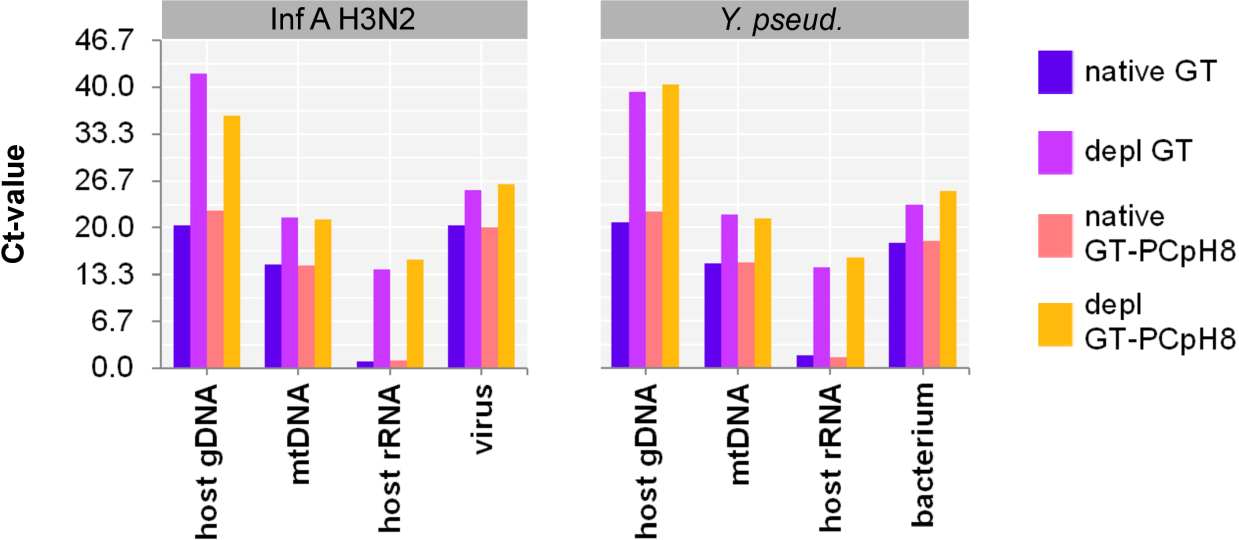


**Supplementary Figure 5.** **Concomitant DNA and RNA extraction by a guanidinium-thiocyanate (GT) compared to a GT‑phenol‑chloroform (GT‑PC) based method for metagenomics analysis.** qPCR results of native and host NA depleted surrogate CSF samples spiked with either **A:** RNA viruses (Inf A H3N2) or **B:** bacteria (*Y. pseud.*), respectively, comparing two different NA extraction approaches: a GT buffer based automated system (MagNA Pure 96, DNA and Viral NA Large Volume Kit, Viral NA Universal LV 1000 3.0.1 run protocol) and a GT‑PC buffer at pH 8 based method with subsequent purification by MagNA Pure 96. Primer-probe targets listed on x‑axis: actin B gene host cell genomic DNA (host gDNA), the mitochondrial NADH-ubiquinone oxidoreductase chain 1 DNA (mtDNA), the eukaryotic 18S ribosomal RNA (host rRNA), the matrix proteins of Influenza A (virus), the putative siderophore biosynthesis protein of *Y.pseud.* (bacterium). The y‑axis shows the cycle threshold value (Ct-value) in steps of 3.33, representing an approximated 10 fold reduction of copy number.

**(A)**

**
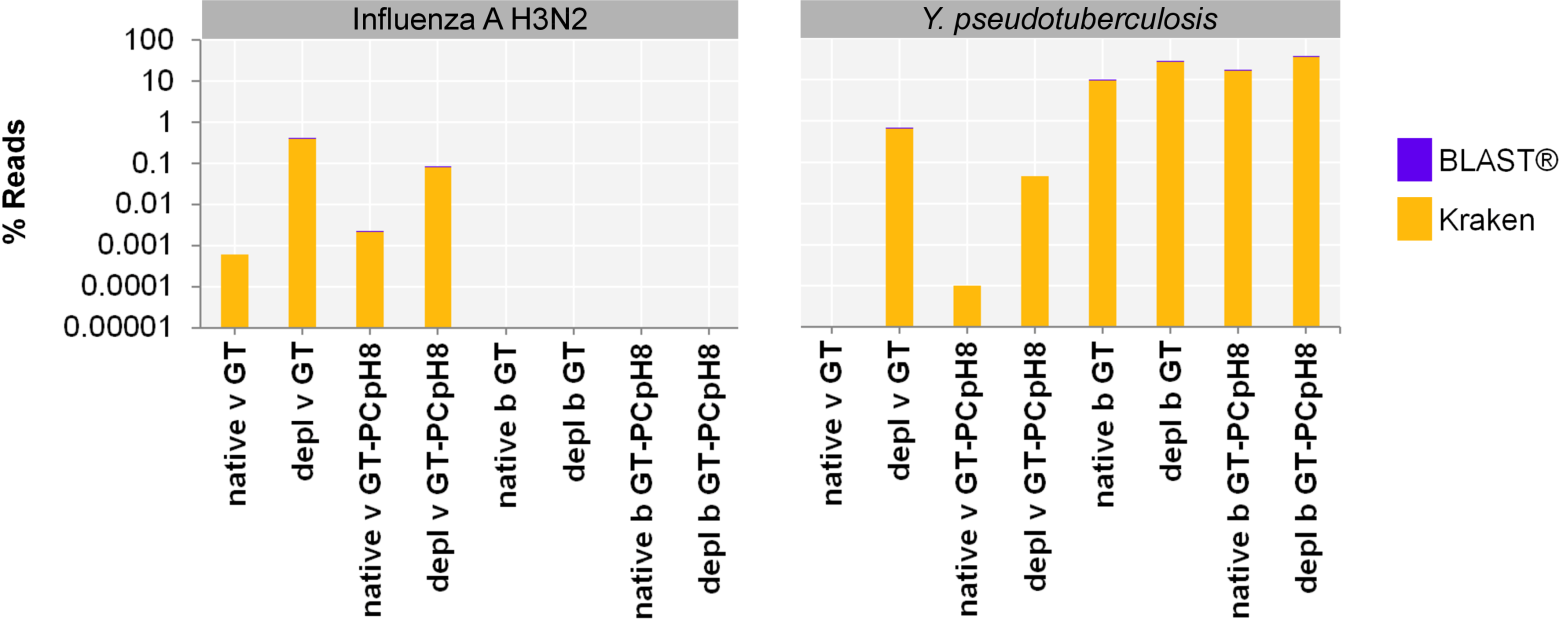
**

**(C)**

**(B)**

**
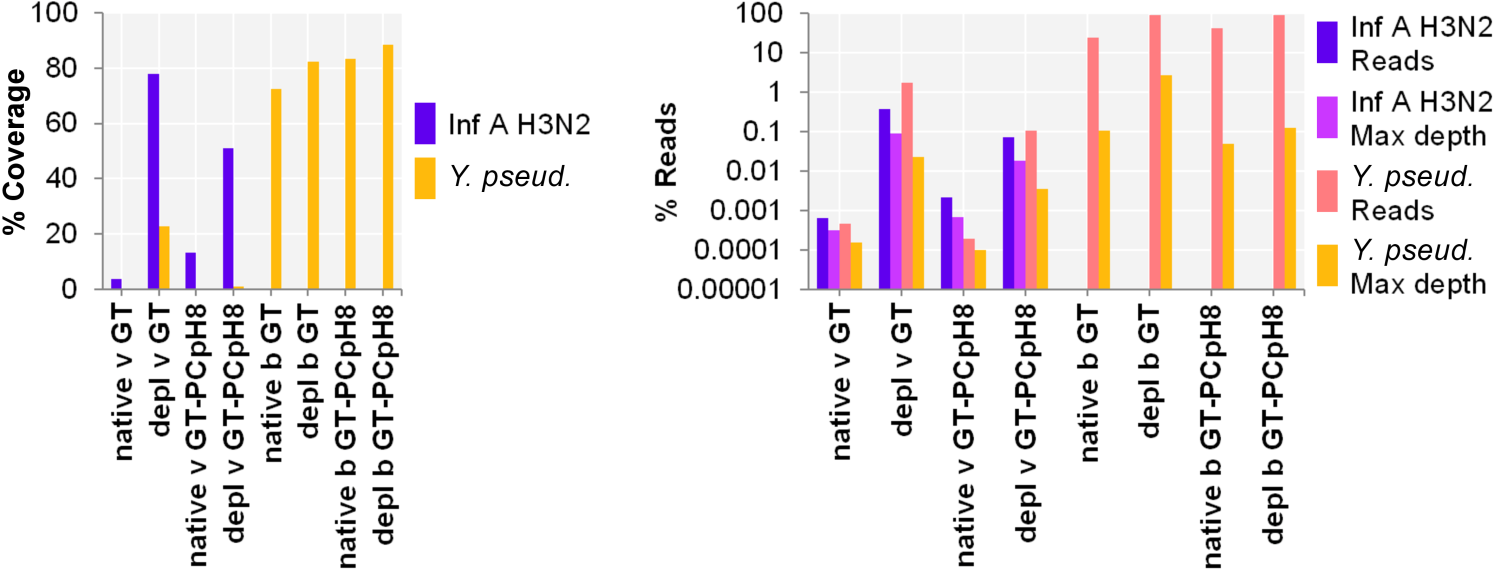
**

**Supplementary Figure 6. Concomitant DNA and RNA extraction by a guanidinium-thiocyanate (GT) compared to a GT‑phenol‑chloroform (GT‑PCpH8) based method for metagenomics analysis.** Sequencing results of native and host NA depleted surrogate CSF samples spiked with either RNA viruses Influenza A virus H3N2 (v; Inf A H3N2) or bacteria *Y. pseudotuberculosis* (b; *Y. pseud.*), respectively, comparing two different NA extraction approaches: a GT buffer based automated system (MagNA Pure 96, DNA and Viral NA Large Volume Kit, Viral NA Universal LV 1000 3.0.1 run protocol) and a GT‑PC buffer at pH 8 based method with subsequent purification by MagNA Pure 96. **A:** Taxonomical classification of reads to Inf A H3N2 and *Y. pseud.* by exact k‑mer mapping (Kraken) to a custom database of human, viruses and bacteria assemblies from RefSeq or Genbank® complemented with local alignment to the NCBI’s nt (BLAST®) of unclassified reads by Kraken. **B:** % Reference sequence coverage and **C:** reads aligned with maximum depth of coverage (Max depth) results of reference sequence alignments (Bowtie2) to respective species. **A,C:** Y‑axis shows the number reads in % respective to the total reads that were taxonomically classified or assigned as unclassified by Kraken in decimal logarithm scale.


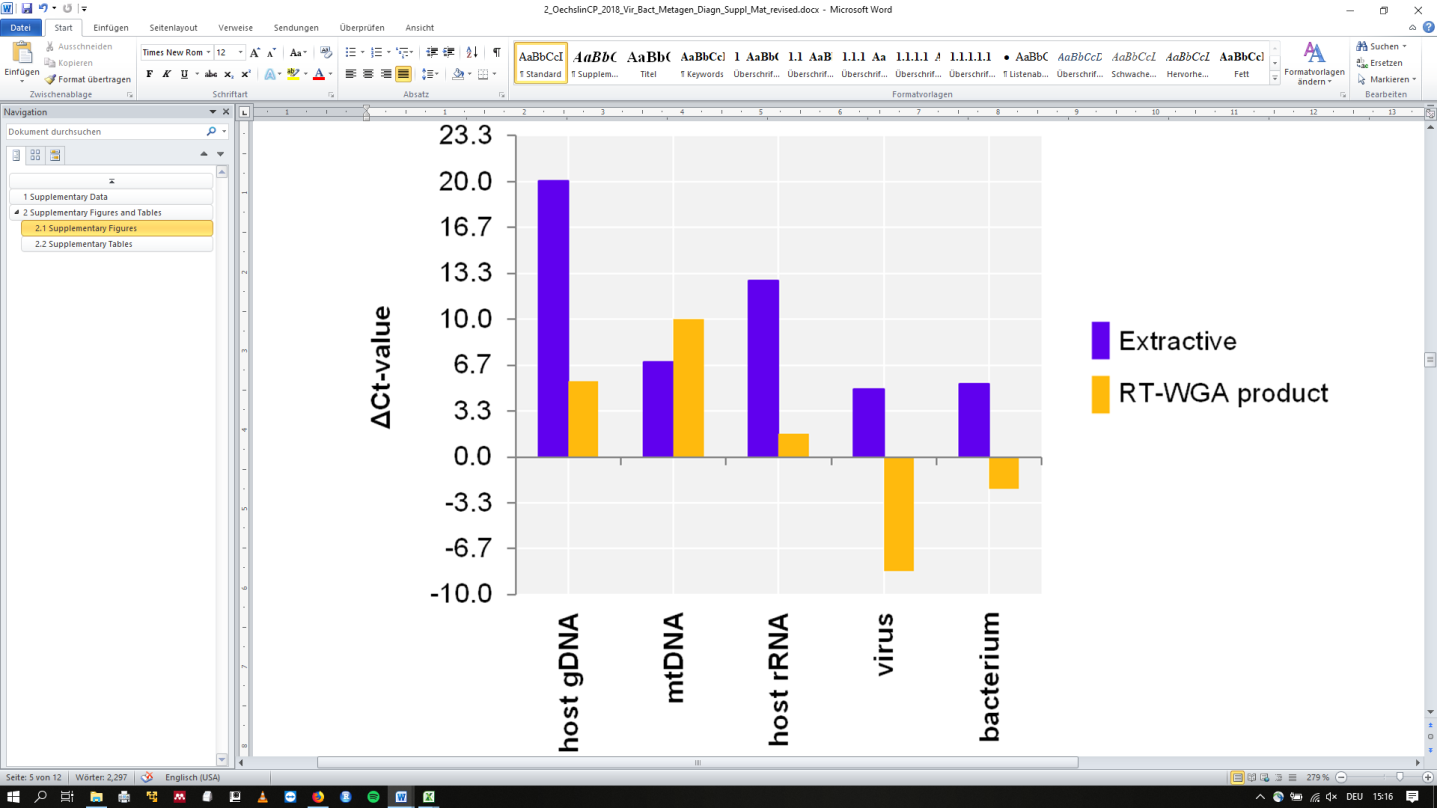


**Supplementary Figure 7. Comparison of total depletion observed in extractives and following RT-WGA** of surrogate CSF samples separately spiked by virus and bacterium. Analysis was performed using qPCR assessing the cycle threshold value (Ct-value) after whole NA extraction and following reverse transcription and whole genome amplification (RT-WGA). Total depletion was calculated as the difference (Δ) between a native and a host NA depleted sample. Primer-probe targets listed on x-axis: the actin B gene host cell genomic DNA (gDNA), the mitochondrial NADH-ubiquinone oxidoreductase chain 1 DNA (mtDNA) and the eukaryotic 18S ribosomal RNA (host rRNA), the matrix proteins of Influenza A virus (virus), the putative siderophore biosynthesis protein of *Y. pseudotuberculosis* (bacterium). The y-axis shows ΔCt-value changes of 3.33 per steps, representing an approximated 10-fold reduction and increase of copy numbers of positive and negative ΔCt-values, respectively.

## Supplementary Tables

| **SUpplementary Table 1 \|** Overview of the preparation and sequencing data of the samples used in this study. | | | | | | | | | | |
| --- | --- | --- | --- | --- | --- | --- | --- | --- | --- | --- |
| **Sample** | **Sample preparation** | **Amplification** | **Size-selection** | **Sequencing system** | **Chip/lane fraction^a^** | **Chip loading (%)** | **Output  (bp)** | **Output  (reads)** |  | **Output  (%)** |
| **Results section: WTA for RNA viruses** | | | | | | | | | | |
| v1to1, Inf A | native | WTA2 | AMPure® XP | HiSeq 2500 V4 | 0.167 | n/a | 3,153,726,125 | 25,229,809 | 33.6 | |
| v1to100, Inf A | native | WTA2 | AMPure® XP | HiSeq 2500 V4 | 0.167 | n/a | 2,826,965,875 | 22,615,727 | 30.2 | |
| v1to10,000, Inf A | native | WTA2 | AMPure® XP | HiSeq 2500 V4 | 1 | n/a | 15,913,645,250 | 127,309,162 | 28.3 | |
| v1to1, Inf A | host NA depl | WTA2 | AMPure® XP | HiSeq 2500 V4 | 0.167 | n/a | 1,569,006,875 | 12,552,055 | 16.7 | |
| v1to100, Inf A | host NA depl | WTA2 | AMPure® XP | HiSeq 2500 V4 | 0.167 | n/a | 2,332,815,875 | 18,662,527 | 24.9 | |
| v1to10,000, Inf A | host NA depl | WTA2 | AMPure® XP | HiSeq 2500 V4 | 1 | n/a | 12,640,771,500 | 101,126,172 | 22.5 | |
| **Results section: WGA for DNA viruses and bacteria** | | | | | | | | | | |
| RT-WGA, Inf A + *Y. pseud.* | host NA depl | RT-WGA | AMPure® XP | Ion Proton™ | Ion PI™, barcoded | 72.0 | 571,543,120 | 4,617,612 | 7.7 | |
| WTA, Inf A + *Y. pseud.* | host NA depl | WTA2 | AMPure® XP | Ion Proton™ | Ion PI™, barcoded | 72.0 | 1,907,301,156 | 16,008,698 | 26.7 | |
| **Results section: Samples of patients (Table 1)** | | | | | | | | | | |
| 1: VZV | native | RT-WGA | LabChip® XT | Ion S5™ | Ion 540™ | 74.0 | 4,890,000,000 | 33,029,801 | 30.5 | |
| 2: VZV | host NA depl | RT-WGA | LabChip® XT | Ion S5™ | Ion 540™ | 27.3 | 1,120,000,000 | 9,840,433 | 25.1 | |
| 3: HSV-1 | native | RT-WGA | LabChip® XT | Ion S5™ | Ion 540™ | 63.3 | 5,440,000,000 | 34,966,102 | 37.8 | |
| 4: HSV-1 | host NA depl | RT-WGA | LabChip® XT | Ion S5™ | Ion 540™ | 40.5 | 1,280,000,000 | 11,486,457 | 19.6 | |
| 5: Enterovirus | native | RT-WGA | LabChip® XT | Ion S5™ | Ion 540™ | 82.3 | 7,160,000,000 | 48,092,069 | 39.9 | |
| 6: Enterovirus | host NA depl | RT-WGA | LabChip® XT | Ion S5™ | Ion 540™ | 38.9 | 1,650,000,000 | 13,393,825 | 23.9 | |
| 7: Enterovirus | native | WTA2 | AMPure® XP | HiSeq 2500 V4 | 0.167 | n/a | 2,418,727,875 | 19,349,823 | 25.8 | |
| 8: Enterovirus | host NA depl | WTA2 | AMPure® XP | HiSeq 2500 V4 | 0.167 | n/a | 1,941,214,625 | 15,529,717 | 20.7 | |
| 9: Enterovirus | host NA depl | RT-WGA | AMPure® XP | Ion S5™ | Ion 540™ | 84.1 | 7,980,000,000 | 62,677,514 | 50.7 | |
| 10: Enterovirus | native | RT-WGA | AMPure® XP | HiSeq 2500 V4 | 0.5 | n/a | 9,917,235,250 | 79,337,882 | 35.3 | |
| 11: Enterovirus | host NA depl | RT-WGA | AMPure® XP | HiSeq 2500 V4 | 0.5 | n/a | 10,258,465,875 | 82,067,727 | 36.5 | |
| *(Continued)* | | | | | | | | | | |
| **SUpplementary Table 1 \|** Continued | | | | | | | | | | |
| **Sample** | **Sample preparation** | **Amplification** | **Size-selection** | **Sequencing system** | **Chip/lane fraction^a^** | **Chip loading (%)** | **Output  (bp)** | **Output  (reads)** |  | **Output  (%)** |
| 12: ß-hemol. A Streptococci | native | RT-WGA | AMPure® XP | HiSeq 2500 V4 | 0.5 | n/a | 17,076,980,625 | 136,615,845 | 60.7 | |
| 13: ß-hemol. A Streptococci | host NA depl | RT-WGA | AMPure® XP | HiSeq 2500 V4 | 0.5 | n/a | 13,966,660,250 | 111,733,282 | 49.7 | |
| 14: JCV | native | RT-WGA | AMPure® XP | HiSeq 2500 V4 | 0.5 | n/a | 5,617,714,500 | 44,941,716 | 20.0 | |
| 15: JCV | host NA depl | RT-WGA | AMPure® XP | HiSeq 2500 V4 | 0.5 | n/a | 4,107,416,875 | 32,859,335 | 14.6 | |
| 16: HSV | native | RT-WGA | AMPure® XP | HiSeq 2500 V4 | 0.5 | n/a | 7,045,225,750 | 56,361,806 | 25.0 | |
| 17: HSV | host NA depl | RT-WGA | AMPure® XP | HiSeq 2500 V4 | 0.5 | n/a | 7,873,648,125 | 62,989,185 | 28.0 | |
| 18: VZV | native | RT-WGA | AMPure® XP | HiSeq 2500 V4 | 0.5 | n/a | 5,089,302,000 | 40,714,416 | 18.1 | |
| 19: VZV | host NA depl | RT-WGA | AMPure® XP | HiSeq 2500 V4 | 0.5 | n/a | 4,528,130,875 | 36,225,047 | 16.1 | |
| 20: 1 Enterovirus | native | RT-WGA | AMPure® XP | HiSeq 2500 V4 | 0.5 | n/a | 7,879,313,500 | 63,034,508 | 28.0 | |
| 21: 1 Enterovirus | host NA depl | RT-WGA | AMPure® XP | HiSeq 2500 V4 | 0.5 | n/a | 4,249,360,000 | 33,994,880 | 15.1 | |
| 22: *Staph. aureus* | native | RT-WGA | AMPure® XP | HiSeq 2500 V4 | 0.5 | n/a | 4,221,415,000 | 33,771,320 | 15.0 | |
| 23: *Staph. aureus* | host NA depl | RT-WGA | AMPure® XP | HiSeq 2500 V4 | 0.5 | n/a | 3,893,571,375 | 31,148,571 | 13.8 | |
| 24: Enterovirus | native | RT-WGA | AMPure® XP | Ion S5™ | Ion 540™ | 82.6 | 5,620,000,000 | 43,390,979 | 34.9 | |
| 25: Enterovirus | host NA depl | RT-WGA | AMPure® XP | Ion S5™ | Ion 540™ | 84.2 | 8,230,000,000 | 60,434,562 | 47.6 | |
| 26: HHV-6 | native | RT-WGA | AMPure® XP | Ion S5™ | Ion 540™ | 75.5 | 6,200,000,000 | 44,144,285 | 38.7 | |
| 27: HHV-6 | host NA depl | RT-WGA | AMPure® XP | Ion S5™ | Ion 540™ | 80.5 | 6,980,000,000 | 49,012,528 | 40.5 | |
| 28: Rhinovirus (nose swab) | native | RT-WGA | AMPure® XP | Ion S5™ | Ion 540™ | 84.3 | 6,420,000,000 | 47,566,509 | 38.4 | |
| 29: Rhinovirus (nose swab) | host NA depl | RT-WGA | AMPure® XP | Ion S5™ | Ion 540™ | 53.3 | 2,920,000,000 | 21,797,225 | 28.0 | |
| 30: ANDV (serum) | native | RT-WGA | AMPure® XP | Ion S5™ | Ion 540™ | 58.5 | 2,810,000,000 | 24,172,858 | 28.1 | |
| 31: ANDV (serum) | host NA depl | RT-WGA | AMPure® XP | Ion S5™ | Ion 540™ | 53.2 | 2,890,000,000 | 23,371,781 | 30.0 | |
| **Results section: Negative controls, contamination and noise** | | | | | | | | | | |
| neg | native | RT-WGA | AMPure® XP | HiSeq 2500 V4 | 0.5 | n/a | 8,810,604,500 | 70,484,836 | 31.3 | |
| neg | host NA depl | RT-WGA | AMPure® XP | HiSeq 2500 V4 | 0.5 | n/a | 4,922,713,500 | 39,381,708 | 17.5 | |
| *(Continued)* | | | | | | | | | | |
| **SUpplementary Table 1 \|** Continued | | | | | | | | | | |
| **Sample** | **Sample preparation** | **Amplification** | **Size-selection** | **Sequencing system** | **Chip/lane fraction^a^** | **Chip loading (%)** | **Output  (bp)** | **Output  (reads)** |  | **Output  (%)** |
| v1to1, Inf A | native | WTA2 | AMPure® XP | Ion S5™ | Ion 530™ | 76.0 | 1,310,000,000 | 6,949,104 | 25.3 | |
| v1to,1 Inf A | host NA depl | WTA2 | AMPure® XP | Ion S5™ | Ion 530™ | 87.4 | 835,000,000 | 4,431,226 | 13.7 | |
| v1to1, Inf A | native | WTA2 | AMPure® XP | HiSeq 2500 V4 | 0.167 | n/a | 3,153,726,125 | 25,229,809 | 33.6 | |
| v1to1, Inf A | host NA depl | WTA2 | AMPure® XP | HiSeq 2500 V4 | 0.167 | n/a | 1,569,006,875 | 12,552,055 | 16.7 | |
| patCSF unknown | native | RT-WGA | AMPure® XP | Ion S5™ | Ion 540™ | 85.0 | 4,940,000,000 | 40,632,454 | 32.5 | |
| nc(H2O) patCSF unknown | native | RT-WGA | AMPure® XP | Ion S5™ | Ion 540™ | 42.2 | 1,470,000,000 | 12,760,260 | 20.8 | |
| patCSF unknown | host NA depl | RT-WGA | AMPure® XP | Ion S5™ | Ion 540™ | 15.5 | 356,000,000 | 4,090,656 | 19.1 | |
| nc(H2O) patCSF unknown | host NA depl | RT-WGA | AMPure® XP | Ion S5™ | Ion 540™ | 10.8 | 79,500,000 | 1,187,674 | 8.4 | |
| nc(PBS) nose swab | native | RT-WGA | AMPure® XP | Ion S5™ | Ion 540™ | 75.9 | 5,680,000,000 | 38,655,386 | 34.6 | |
| nc(PBS) nose swab | host NA depl | RT-WGA | AMPure® XP | Ion S5™ | Ion 540™ | 53.3 | 90,700,000 | 21,797,225 | 28.0 | |
| *^a^Ion 530™ 400 base-pair (bp) theoretical output: 3,000,000,000 bp, 15,000,000 reads; Ion PI™/Ion 540™ theoretical output: 10,000,000,000 bp, 60,000,000 reads; Illumina HiSeq2500 V4 2x125 bp 1 lane theoretical output: 56,250,000,000 bp, 450,000,000 reads* | | | | | | | | | | |

| **SUpplementary Table 2 \|** Overview of the trimming output and the rate of taxonomical classification by Kraken/BLAST® of the samples used in this study. | | | | | | | | | | | | | | |
| --- | --- | --- | --- | --- | --- | --- | --- | --- | --- | --- | --- | --- | --- | --- |
| **Sample** | **Sample preparation** | **Sequencing  system** | | **Trimmed (reads)** | **Trimmed (%)** |  | **Kraken (reads)** | |  | | **BLAST® (reads)** | |  | **Kraken/BLAST® (%)** |
| **Results section: WTA for RNA viruses** | | | | | | | | | | | | | | |
| v1to1, Inf A | native | HiSeq 2500 V4 | 18,080,432 | | 71.7 | 7,539,553 | |  | | 74,668 | |  | | 42.1 |
| v1to100, Inf A | native | HiSeq 2500 V4 | 16,706,907 | | 73.9 | 7,238,462 | |  | | 72,693 | |  | | 43.8 |
| v1to10,000, Inf A | native | HiSeq 2500 V4 | 96,699,484 | | 76.0 | 49,935,856 | |  | | 579,834 | |  | | 52.2 |
| v1to1, Inf A | host NA depl | HiSeq 2500 V4 | 9,818,536 | | 78.2 | 2,507,910 | |  | | 24,740 | |  | | 25.8 |
| v1to100, Inf A | host NA depl | HiSeq 2500 V4 | 13,991,346 | | 75.0 | 3,708,703 | |  | | 56,659 | |  | | 26.9 |
| v1to10,000, Inf A | host NA depl | HiSeq 2500 V4 | 79,035,988 | | 78.2 | 22,548,182 | |  | | 421,387 | |  | | 29.1 |
| **Results section: WGA for DNA viruses and bacteria** | | | | | | | | | | | | | | |
| RT-WGA, Inf A + *Y. pseud.* | host NA depl | Ion Proton™ | 3,716,911 | | 80.5 | 174,946 | |  | | 53,884 | |  | | 6.2 |
| WTA, Inf A + *Y. pseud.* | host NA depl | Ion Proton™ | 9,130,255 | | 57.0 | 7,801,790 | |  | | 228,765 | |  | | 88.0 |
| **Results section: Samples of patients (Table 1)** | | | | | | | | | | | | | | |
| 1: VZV | native | Ion S5™ | 8,230,453 | | 24.9 | 7,049,169 | |  | | 217,349 | |  | | 88.3 |
| 2: VZV | host NA depl | Ion S5™ | 2,823,389 | | 28.7 | 2,017,944 | |  | | 104,382 | |  | | 75.2 |
| 3: HSV-1 | native | Ion S5™ | 7,056,116 | | 20.2 | 5,775,896 | |  | | 177,423 | |  | | 84.4 |
| 4: HSV-1 | host NA depl | Ion S5™ | 2,264,987 | | 19.7 | 902,555 | |  | | 287,874 | |  | | 52.6 |
| 5: Enterovirus | native | Ion S5™ | 8,458,201 | | 17.6 | 6,568,504 | |  | | 256,219 | |  | | 80.7 |
| 6: Enterovirus | host NA depl | Ion S5™ | 3,388,228 | | 25.3 | 2,460,927 | |  | | 128,304 | |  | | 76.4 |
| 7: Enterovirus | native | HiSeq 2500 V4 | 12,135,462 | | 62.7 | 4,414,327 | |  | | 58,137 | |  | | 36.9 |
| 8: Enterovirus | host NA depl | HiSeq 2500 V4 | 10,773,001 | | 69.4 | 3,425,442 | |  | | 87,585 | |  | | 32.6 |
| 9: Enterovirus | host NA depl | Ion S5™ | 12,806,773 | | 20.4 | 8,065,906 | |  | | 1,314,093 | |  | | 73.2 |
| 10: Enterovirus | native | HiSeq 2500 V4 | 64,362,407 | | 81.1 | 9,297,727 | |  | | 1,437,850 | |  | | 16.7 |
| 11: Enterovirus | host NA depl | HiSeq 2500 V4 | 67,504,348 | | 82.3 | 37,940,492 | |  | | 2,909,094 | |  | | 60.5 |
| *(Continued)* | | | | | | | | | | | | | | |

| **SUpplementary Table 2 \|** Continued | | | | | | | | | | | | | | |
| --- | --- | --- | --- | --- | --- | --- | --- | --- | --- | --- | --- | --- | --- | --- |
| **Sample** | **Sample preparation** | **Sequencing  system** | | **Trimmed (reads)** | **Trimmed (%)** |  | **Kraken (reads)** | |  | | **BLAST® (reads)** | |  | **Kraken/BLAST® (%)** |
| 12: ß-hemol. A Streptococci | native | HiSeq 2500 V4 | 116,130,903 | | 85.0 | 11,184,852 | |  | | 1,145,447 | |  | | 10.6 |
| 13: ß-hemol. A Streptococci | host NA depl | HiSeq 2500 V4 | 96,442,185 | | 86.3 | 10,681,604 | |  | | 5,539,341 | |  | | 16.8 |
| 14: JCV | native | HiSeq 2500 V4 | 36,063,475 | | 80.2 | 32,258,940 | |  | | 683,759 | |  | | 91.3 |
| 15: JCV | host NA depl | HiSeq 2500 V4 | 27,342,432 | | 83.2 | 23,590,562 | |  | | 576,292 | |  | | 88.4 |
| 16: HSV | native | HiSeq 2500 V4 | 46,187,640 | | 81.9 | 18,589,644 | |  | | 821,263 | |  | | 42.0 |
| 17: HSV | host NA depl | HiSeq 2500 V4 | 52,839,670 | | 83.9 | 8,858,871 | |  | | 1,696,298 | |  | | 20.0 |
| 18: VZV | native | HiSeq 2500 V4 | 32,040,038 | | 78.7 | 21,124,279 | |  | | 702,664 | |  | | 68.1 |
| 19: VZV | host NA depl | HiSeq 2500 V4 | 30,538,370 | | 84.3 | 27,090,563 | |  | | 570,664 | |  | | 90.6 |
| 20: 1 Enterovirus | native | HiSeq 2500 V4 | 53,537,573 | | 84.9 | 8,271,884 | |  | | 1,285,066 | |  | | 17.9 |
| 21: 1 Enterovirus | host NA depl | HiSeq 2500 V4 | 28,262,000 | | 83.1 | 11,638,341 | |  | | 1,276,389 | |  | | 45.7 |
| 22: *Staph. aureus* | native | HiSeq 2500 V4 | 26,614,160 | | 78.8 | 22,153,005 | |  | | 563,380 | |  | | 85.4 |
| 23: *Staph. aureus* | host NA depl | HiSeq 2500 V4 | 25,475,172 | | 81.8 | 22,268,496 | |  | | 515,377 | |  | | 89.4 |
| 24: Enterovirus | native | Ion S5™ | 9,469,376 | | 21.8 | 8,751,820 | |  | | 128,350 | |  | | 93.8 |
| 25: Enterovirus | host NA depl | Ion S5™ | 12,661,997 | | 21.0 | 11,038,294 | |  | | 223,198 | |  | | 88.9 |
| 26: HHV-6 | native | Ion S5™ | 13,782,537 | | 31.2 | 12,772,319 | |  | | 170,181 | |  | | 93.9 |
| 27: HHV-6 | host NA depl | Ion S5™ | 13,569,093 | | 27.7 | 12,679,133 | |  | | 170,579 | |  | | 94.7 |
| 28: Rhinovirus (nose swab) | native | Ion S5™ | 10,458,388 | | 22.0 | 9,679,806 | |  | | 172,166 | |  | | 94.2 |
| 29: Rhinovirus (nose swab) | host NA depl | Ion S5™ | 11,288,055 | | 51.8 | 8,195,591 | |  | | 263,989 | |  | | 74.9 |
| 30: ANDV (serum) | native | Ion S5™ | 4,637,740 | | 19.2 | 4,102,859 | |  | | 74,613 | |  | | 90.1 |
| 31: ANDV (serum) | host NA depl | Ion S5™ | 7,830,397 | | 33.5 | 5,790,707 | |  | | 193,320 | |  | | 76.4 |
| **Results section: Negative controls, contamination and noise** | | | | | | | | | | | | | | |
| neg | native | HiSeq 2500 V4 | 57,639,193 | | 81.8 | 21,787,754 | |  | | 947,303 | |  | | 39.4 |
| neg | host NA depl | HiSeq 2500 V4 | 33,275,778 | | 84.5 | 28,876,832 | |  | | 601,258 | |  | | 88.6 |
| *(Continued)* | | | | | | | | | | | | | | |
| **SUpplementary Table 2 \|** Continued | | | | | | | | | | | | | | |
| **Sample** | **Sample preparation** | **Sequencing  system** | | **Trimmed (reads)** | **Trimmed (%)** |  | **Kraken (reads)** | |  | | **BLAST® (reads)** | |  | **Kraken/BLAST® (%)** |
| v1to1, Inf A | native | Ion S5™ | 4,374,995 | | 63.0 | 3,760,103 | |  | | 73,869 | |  | | 87.6 |
| v1to,1 Inf A | host NA depl | Ion S5™ | 1,494,911 | | 33.7 | 1,198,377 | |  | | 33,942 | |  | | 82.4 |
| v1to1, Inf A | native | HiSeq 2500 V4 | 18,080,432 | | 71.7 | 7,539,553 | |  | | 74,668 | |  | | 42.1 |
| v1to1, Inf A | host NA depl | HiSeq 2500 V4 | 9,818,536 | | 78.2 | 2,507,910 | |  | | 24,740 | |  | | 25.8 |
| patCSF unknown | native | Ion S5™ | 9,444,820 | | 23.2 | 8,721,750 | |  | | 132,464 | |  | | 93.7 |
| nc(H2O) patCSF unknown | native | Ion S5™ | 3,512,492 | | 27.5 | 2,072,024 | |  | | 146,040 | |  | | 63.1 |
| patCSF unknown | host NA depl | Ion S5™ | 1,213,309 | | 29.7 | 401,472 | |  | | 31,432 | |  | | 35.7 |
| nc(H2O) patCSF unknown | host NA depl | Ion S5™ | 284,392 | | 23.9 | 20,817 | |  | | 2,164 | |  | | 8.1 |
| nc(PBS) nose swab | native | Ion S5™ | 13,628,913 | | 35.3 | 3,132,710 | |  | | 950,040 | |  | | 30.0 |
| nc(PBS) nose swab | host NA depl | Ion S5™ | 11,288,055 | | 51.8 | 44,747 | |  | | 13,099 | |  | | 0.5 |
|  | | | | | | | | | | | | | | |

| **SUpplementary Table 3 \|** List of genome accession numbers used in this study for separate reference alignment by Bowtie2. | |
| --- | --- |
| **Species** | **Accession number** |
| Human enterovirus A | NC_001612.1 |
| Human enterovirus B | NC_001472.1 |
| Human enterovirus C | NC_002058.3 |
| Human enterovirus D | NC_001430.1 |
| Human rhinovirus A | A10937.1 |
| Human rhinovirus B | NC_001490.1 |
| Human rhinovirus C | NC_009996.1 |
| Human herpesvirus 1/Herpes simplex virus 1 | NC_001806.2 |
| Human herpesvirus 2/Herpes simplex virus 2 | NC_001798.2 |
| Human herpesvirus 3/Varicella Zoster virus | NC_001348.1 |
| Human herpesvirus 6 | KY239023.1 |
| Human herpesvirus 6A | NC_001664.2 |
| Human herpesvirus 6B | NC_000898.1 |
| JC polyomavirus | NC_001699.1 |
| Andes orthohantavirus Chile | NC_003466.1 |
|  | NC_003467.2 |
|  | NC_003468.2 |
| Influenza A H3N2 | NC_007366.1 |
|  | NC_007367.1 |
|  | NC_007368.1 |
|  | NC_007369.1 |
|  | NC_007370.1 |
|  | NC_007371.1 |
|  | NC_007372.1 |
|  | NC_007373.1 |
| *Staphylococcus aureus* | NC_007795.1 |
| *Streptococcus pyogenes* | NC_002737.2 |
| *Streptococcus dysgalactiae subsp. equisimilis* | NC_019042.1 |
| *Streptococcus anginosus* | NC_022239.1 |
| *Streptococcus constellatus subsp. pharyngis* | NC_022238.1 |
| *Streptococcus intermedius* | NC_022246.1 |
| *Yersinia pseudotuberculosis* | NZ_CP008943.1 |

| **SUpplementary Table 4 \|** Taxonomic profiles of Kraken after noise reduction of the surrogate CSF samples spiked with three 100‑fold dilutions of virus spiking suspension (***Influenza A virus***). | | | | |
| --- | --- | --- | --- | --- |
| **Taxonomic profile of bacteria and viruses species** | |  | **Taxonomic profile of viruses species only** | |
|  |  |  |  |  |
| **native v1to1** |  |  |  |  |
| **Species** | **% Reads** |  | **Species** | **% Reads** |
| Homo sapiens | 9.8E+01 |  | ***Influenza A virus*** | ***1.4E-04*** |
| ***Influenza A virus*** | ***1.4E-04*** |  |  |  |
| Xanthomonas alfalfae | 1.3E-04 |  |  |  |
| Bacillus thuringiensis | 1.0E-04 |  |  |  |
| Bacillus cereus | 5.2E-05 |  |  |  |
| Cutibacterium acnes | 3.9E-05 |  |  |  |
| Enterobacter cloacae | 2.6E-05 |  |  |  |
| Haemophilus parainfluenzae | 2.6E-05 |  |  |  |
|  |  |  |  |  |
| **native v1to100** |  |  |  |  |
| **Species** | **% Reads** |  | **Species** | **% Reads** |
| Homo sapiens | 9.8E+01 |  | ***Influenza A virus*** | ***9.5E-05*** |
| Cutibacterium acnes | 1.8E-04 |  |  |  |
| ***Influenza A virus*** | ***9.5E-05*** |  |  |  |
| Bacillus thuringiensis | 6.8E-05 |  |  |  |
|  |  |  |  |  |
| **native v1to10,000** |  |  |  |  |
| **Species** | **% Reads** |  | **Species** | **% Reads** |
| Homo sapiens | 9.7E+01 |  | Bacillus phage Phrodo | 1.9E-06 |
| Bacillus thuringiensis | 2.2E-04 |  | Pandoravirus inopinatum | 1.9E-06 |
| Cutibacterium acnes | 1.1E-04 |  | Mollivirus sibericum | 1.9E-06 |
| Xanthomonas alfalfae | 3.7E-05 |  |  |  |
| Burkholderia pseudomallei | 2.3E-05 |  |  |  |
| Pseudomonas synxantha | 2.1E-05 |  |  |  |
| Borrelia crocidurae | 2.1E-05 |  |  |  |
| Pseudomonas frederiksbergensis | 1.6E-05 |  |  |  |
| Chamaesiphon minutus | 1.2E-05 |  |  |  |
| Janthinobacterium sp. 1_2014MBL_MicDiv | 1.2E-05 |  |  |  |
| Acidovorax sp. JS42 | 7.8E-06 |  |  |  |
| Borrelia hermsii | 7.8E-06 |  |  |  |
| *(Continued)* | | | | |

| **SUpplementary Table 4 \|** Continued | | | | |
| --- | --- | --- | --- | --- |
| **Taxonomic profile of bacteria and viruses species** | |  | **Taxonomic profile of viruses species only** | |
|  |  |  |  |  |
| **host NA depleted v1to1** |  |  |  |  |
| **Species** | **% Reads** |  | **Species** | **% Reads** |
| Homo sapiens | 9.5E+01 |  | ***Influenza A virus*** | ***1.2E+00*** |
| ***Influenza A virus*** | ***1.2E+00*** |  | Brome mosaic virus | 3.9E-04 |
| Cutibacterium acnes | 3.0E-02 |  |  |  |
| Bacillus cereus | 2.4E-02 |  |  |  |
| Yersinia pseudotuberculosis | 6.8E-03 |  |  |  |
| Escherichia coli | 6.0E-03 |  |  |  |
| Moraxella osloensis | 4.7E-03 |  |  |  |
| Staphylococcus epidermidis | 3.8E-03 |  |  |  |
| Streptococcus gordonii | 3.5E-03 |  |  |  |
| Pseudomonas sihuiensis | 3.2E-03 |  |  |  |
| Streptococcus mitis | 2.6E-03 |  |  |  |
| Acidovorax sp. KKS102 | 2.2E-03 |  |  |  |
| Corynebacterium kroppenstedtii | 1.6E-03 |  |  |  |
| Haemophilus parainfluenzae | 1.5E-03 |  |  |  |
| Acinetobacter johnsonii | 1.3E-03 |  |  |  |
| Acidovorax sp. RAC01 | 1.3E-03 |  |  |  |
| Psychrobacter urativorans | 1.2E-03 |  |  |  |
| Corynebacterium ureicelerivorans | 1.0E-03 |  |  |  |
| Gemmata sp. SH-PL17 | 9.8E-04 |  |  |  |
| Lawsonella clevelandensis | 9.4E-04 |  |  |  |
| Stenotrophomonas maltophilia | 9.0E-04 |  |  |  |
| Lactobacillus curvatus | 8.6E-04 |  |  |  |
| Micrococcus luteus | 7.8E-04 |  |  |  |
| Veillonella parvula | 7.4E-04 |  |  |  |
| Corynebacterium sp. ATCC 6931 | 7.4E-04 |  |  |  |
| Comamonas testosteroni | 7.4E-04 |  |  |  |
| Streptococcus sp. oral taxon 064 | 7.0E-04 |  |  |  |
| Pseudomonas pseudoalcaligenes | 6.6E-04 |  |  |  |
| Staphylococcus warneri | 6.3E-04 |  |  |  |
| Acinetobacter baumannii | 6.3E-04 |  |  |  |
| Sphingomonas panacis | 6.3E-04 |  |  |  |
| Ezakiella massiliensis | 5.9E-04 |  |  |  |
| Streptococcus pneumoniae | 5.5E-04 |  |  |  |
| Acidovorax sp. JS42 | 5.5E-04 |  |  |  |
| Sphingomonas hengshuiensis | 5.5E-04 |  |  |  |
| *(Continued)* | | | | |

| **SUpplementary Table 4 \|** Continued | | | | |
| --- | --- | --- | --- | --- |
| **Taxonomic profile of bacteria and viruses species** | |  | **Taxonomic profile of viruses species only** | |
|  |  |  |  |  |
| **host NA depleted v1to100** |  |  |  |  |
| **Species** | **% Reads** |  | **Species** | **% Reads** |
| Homo sapiens | 9.4E+01 |  | Mongoose associated gemykibivirus 1 | 2.9E-02 |
| Bacillus cereus | 1.1E-01 |  | **Influenza A virus** | **1.5E-02** |
| Kocuria palustris | 5.8E-02 |  | Mongoose feces-associated gemycircularvirus c | 3.4E-03 |
| Cutibacterium acnes | 5.7E-02 |  | Bovine associated gemykibivirus 1 | 8.1E-04 |
| Lactobacillus delbrueckii | 4.6E-02 |  | Escherichia virus Lambda | 2.1E-04 |
| Brevibacterium linens | 4.1E-02 |  | Salmonella phage FSL SP-058 | 1.6E-04 |
| Mongoose associated gemykibivirus 1 | 2.9E-02 |  | Brome mosaic virus | 1.3E-04 |
| Streptococcus thermophilus | 2.4E-02 |  |  |  |
| Staphylococcus epidermidis | 1.6E-02 |  |  |  |
| ***Influenza A virus*** | ***1.5E-02*** |  |  |  |
| Corynebacterium casei | 1.3E-02 |  |  |  |
| Escherichia coli | 1.1E-02 |  |  |  |
| Streptococcus mitis | 1.0E-02 |  |  |  |
| Moraxella osloensis | 8.9E-03 |  |  |  |
| Brevibacterium sandarakinum | 8.1E-03 |  |  |  |
| Pseudomonas sihuiensis | 6.7E-03 |  |  |  |
| Corynebacterium kroppenstedtii | 6.4E-03 |  |  |  |
| Anaerococcus prevotii | 5.0E-03 |  |  |  |
| Acidovorax sp. KKS102 | 5.0E-03 |  |  |  |
| Streptococcus sanguinis | 4.9E-03 |  |  |  |
| Actinomyces oris | 4.7E-03 |  |  |  |
| Yersinia pseudotuberculosis | 4.6E-03 |  |  |  |
| Halomonas huangheensis | 4.4E-03 |  |  |  |
| [Haemophilus] ducreyi | 4.0E-03 |  |  |  |
| Streptococcus pneumoniae | 4.0E-03 |  |  |  |
| Streptococcus gordonii | 3.9E-03 |  |  |  |
| Halomonas sp. HL-93 | 3.7E-03 |  |  |  |
| Mongoose feces-associated gemycircularvirus c | 3.4E-03 |  |  |  |
| Haemophilus parainfluenzae | 3.3E-03 |  |  |  |
| Brachybacterium faecium | 3.2E-03 |  |  |  |
| Streptococcus salivarius | 3.1E-03 |  |  |  |
| Micrococcus luteus | 3.1E-03 |  |  |  |
| Streptococcus sp. VT 162 | 2.9E-03 |  |  |  |
| Corynebacterium aurimucosum | 2.9E-03 |  |  |  |
| Acinetobacter johnsonii | 2.8E-03 |  |  |  |
| Corynebacterium variabile | 2.7E-03 |  |  |  |
| *(Continued)* | | | | |
| **SUpplementary Table 4 \|** Continued | | | | |
| **Taxonomic profile of bacteria and viruses species** | |  | **Taxonomic profile of viruses species only** | |
|  |  |  |  |  |
| **host NA depleted v1to100** (continued) |  |  |  |  |
| **Species** | **% Reads** |  | **Species** | **% Reads** |
| Massilia sp. WG5 | 2.7E-03 |  |  |  |
| Prevotella denticola | 2.6E-03 |  |  |  |
| Streptococcus sp. oral taxon 064 | 2.5E-03 |  |  |  |
| Methylobacterium populi | 2.5E-03 |  |  |  |
| Lawsonella clevelandensis | 2.4E-03 |  |  |  |
| Acidovorax sp. RAC01 | 2.3E-03 |  |  |  |
| Brevibacterium siliguriense | 2.2E-03 |  |  |  |
| Rothia mucilaginosa | 2.2E-03 |  |  |  |
| Veillonella parvula | 2.1E-03 |  |  |  |
| Campylobacter gracilis | 2.1E-03 |  |  |  |
| Streptococcus oralis | 2.0E-03 |  |  |  |
| Streptococcus pseudopneumoniae | 1.9E-03 |  |  |  |
| Corynebacterium glyciniphilum | 1.9E-03 |  |  |  |
| Pseudomonas pseudoalcaligenes | 1.8E-03 |  |  |  |
| Chromohalobacter salexigens | 1.7E-03 |  |  |  |
| Finegoldia magna | 1.7E-03 |  |  |  |
| Corynebacterium genitalium | 1.7E-03 |  |  |  |
| Corynebacterium ureicelerivorans | 1.6E-03 |  |  |  |
| Saccharomonospora viridis | 1.6E-03 |  |  |  |
| Hymenobacter sp. PAMC 26554 | 1.5E-03 |  |  |  |
| Staphylococcus saprophyticus | 1.5E-03 |  |  |  |
| Mycobacterium simiae | 1.5E-03 |  |  |  |
| Acinetobacter venetianus | 1.4E-03 |  |  |  |
| Acinetobacter baumannii | 1.4E-03 |  |  |  |
| Corynebacterium simulans | 1.3E-03 |  |  |  |
| Corynebacterium camporealensis | 1.3E-03 |  |  |  |
| Halomonas chromatireducens | 1.3E-03 |  |  |  |
| Thermomonospora curvata | 1.3E-03 |  |  |  |
| Corynebacterium singulare | 1.2E-03 |  |  |  |
| Corynebacterium sp. ATCC 6931 | 1.2E-03 |  |  |  |
| Fusobacterium nucleatum | 1.2E-03 |  |  |  |
| Corynebacterium atypicum | 1.1E-03 |  |  |  |
| Pseudomonas mendocina | 1.1E-03 |  |  |  |
| Prevotella melaninogenica | 1.1E-03 |  |  |  |
| Candidatus Aquiluna sp. UB-MaderosW2red | 1.1E-03 |  |  |  |
| Variovorax paradoxus | 1.1E-03 |  |  |  |
| Streptococcus sp. oral taxon 431 | 1.0E-03 |  |  |  |
| *(Continued)* | | | | |
| **SUpplementary Table 4 \|** Continued | | | | |
| **Taxonomic profile of bacteria and viruses species** | |  | **Taxonomic profile of viruses species only** | |
|  |  |  |  |  |
| **host NA depleted v1to100** (continued) |  |  |  |  |
| **Species** | **% Reads** |  | **Species** | **% Reads** |
| Francisella tularensis | 1.0E-03 |  |  |  |
| Massilia sp. NR 4-1 | 1.0E-03 |  |  |  |
|  |  |  |  |  |
| **host NA depleted v1to10,000** |  |  |  |  |
| **Species** | **% Reads** |  | **Species** | **% Reads** |
| Homo sapiens | 9.4E+01 |  | Escherichia virus Lambda | 2.0E-04 |
| Yersinia pseudotuberculosis | 3.6E-02 |  | Choristoneura occidentalis granulovirus | 1.8E-04 |
| Bacillus cereus | 3.4E-02 |  | Klebsiella phage vB_KpnM_KpV477 | 1.5E-04 |
| Cutibacterium acnes | 2.9E-02 |  | ***Influenza A virus*** | ***1.3E-04*** |
| Moraxella osloensis | 9.5E-03 |  | Salmonella phage phiSG-JL2 | 1.2E-04 |
| Escherichia coli | 7.7E-03 |  | Klebsiella virus 1513 | 1.1E-04 |
| Streptococcus mitis | 6.5E-03 |  | Simbu orthobunyavirus | 1.1E-04 |
| Pseudomonas sihuiensis | 5.3E-03 |  | Klebsiella virus PKO111 | 8.9E-05 |
| Staphylococcus epidermidis | 4.5E-03 |  | Klebsiella virus JD18 | 8.1E-05 |
| Acidovorax sp. KKS102 | 4.2E-03 |  | Klebsiella virus Sushi | 6.4E-05 |
| Acidovorax sp. RAC01 | 2.7E-03 |  | Enterobacteria phage T3 | 6.4E-05 |
| Lactobacillus crispatus | 2.4E-03 |  | Klebsiella virus KP36 | 3.0E-05 |
| Actinomyces oris | 2.3E-03 |  | Staphylococcus phage StB20 | 2.6E-05 |
| Haemophilus parainfluenzae | 2.1E-03 |  | Klebsiella phage K64-1 | 2.6E-05 |
| Streptococcus pneumoniae | 1.7E-03 |  |  |  |
| Acinetobacter johnsonii | 1.7E-03 |  |  |  |
| Acinetobacter venetianus | 1.6E-03 |  |  |  |
| Acinetobacter baumannii | 1.5E-03 |  |  |  |
| Pseudomonas pseudoalcaligenes | 1.4E-03 |  |  |  |
| Streptococcus sanguinis | 1.4E-03 |  |  |  |
| Corynebacterium kroppenstedtii | 1.3E-03 |  |  |  |
| Thermus scotoductus | 1.3E-03 |  |  |  |
| Micrococcus luteus | 1.2E-03 |  |  |  |
| Bacteroides vulgatus | 1.2E-03 |  |  |  |
| Ralstonia pickettii | 1.1E-03 |  |  |  |
| Klebsiella pneumoniae | 1.1E-03 |  |  |  |
| Streptococcus gordonii | 1.0E-03 |  |  |  |
| Pseudomonas mendocina | 8.5E-04 |  |  |  |
| Pseudomonas aeruginosa | 8.2E-04 |  |  |  |
| Rothia dentocariosa | 8.1E-04 |  |  |  |
| Acidovorax sp. JS42 | 7.8E-04 |  |  |  |
| *(Continued)* | | | | |
| **SUpplementary Table 4 \|** Continued | | | | |
| **Taxonomic profile of bacteria and viruses species** | |  | **Taxonomic profile of viruses species only** | |
|  |  |  |  |  |
| **host NA depleted v1to10,000** (continued) |  |  |  |  |
| **Species** | **% Reads** |  | **Species** | **% Reads** |
| Variovorax paradoxus | 7.7E-04 |  |  |  |
| Staphylococcus haemolyticus | 7.5E-04 |  |  |  |
| Rothia mucilaginosa | 7.3E-04 |  |  |  |
| Stenotrophomonas maltophilia | 7.1E-04 |  |  |  |
| Agrobacterium rhizogenes | 7.0E-04 |  |  |  |
| Agrobacterium tumefaciens | 6.9E-04 |  |  |  |
| Comamonas testosteroni | 6.2E-04 |  |  |  |
| Agrobacterium fabrum | 5.7E-04 |  |  |  |
| Streptococcus sp. VT 162 | 5.4E-04 |  |  |  |
| Staphylococcus capitis | 5.3E-04 |  |  |  |
| Gemmata sp. SH-PL17 | 5.2E-04 |  |  |  |
| Fusobacterium nucleatum | 5.1E-04 |  |  |  |
| Ralstonia insidiosa | 5.1E-04 |  |  |  |
| Streptococcus sp. A12 | 4.9E-04 |  |  |  |
| Corynebacterium singulare | 4.7E-04 |  |  |  |
| Streptococcus pseudopneumoniae | 4.7E-04 |  |  |  |
| Veillonella parvula | 4.6E-04 |  |  |  |
| Alicycliphilus denitrificans | 4.5E-04 |  |  |  |
| Bradyrhizobium icense | 4.4E-04 |  |  |  |
| Delftia tsuruhatensis | 4.3E-04 |  |  |  |
| Aerococcus viridans | 4.2E-04 |  |  |  |
| Staphylococcus saprophyticus | 4.2E-04 |  |  |  |
| Leclercia adecarboxylata | 4.0E-04 |  |  |  |
| Prevotella melaninogenica | 4.0E-04 |  |  |  |
| Comamonas aquatica | 4.0E-04 |  |  |  |
| Streptococcus sp. I-P16 | 3.8E-04 |  |  |  |
| Novosphingobium aromaticivorans | 3.7E-04 |  |  |  |
| Acinetobacter sp. NCu2D-2 | 3.6E-04 |  |  |  |
| Pseudomonas fluorescens | 3.6E-04 |  |  |  |
| Acinetobacter lwoffii | 3.4E-04 |  |  |  |
| Haemophilus influenzae | 3.4E-04 |  |  |  |
| Sinorhizobium sp. RAC02 | 3.4E-04 |  |  |  |
| Oligotropha carboxidovorans | 3.4E-04 |  |  |  |
| Streptococcus sp. oral taxon 064 | 3.4E-04 |  |  |  |
| Yersinia pestis | 3.3E-04 |  |  |  |
| Yersinia similis | 3.2E-04 |  |  |  |
| Acinetobacter sp. TTH0-4 | 3.2E-04 |  |  |  |
| *(Continued)* | | | | |
| **SUpplementary Table 4 \|** Continued | | | | |
| **Taxonomic profile of bacteria and viruses species** | |  | **Taxonomic profile of viruses species only** | |
|  |  |  |  |  |
| **host NA depleted v1to10,000** (continued) |  |  |  |  |
| **Species** | **% Reads** |  | **Species** | **% Reads** |
| Stenotrophomonas rhizophila | 3.2E-04 |  |  |  |
| Staphylococcus warneri | 3.2E-04 |  |  |  |
| Hydrogenophaga sp. PBC | 3.1E-04 |  |  |  |
| Streptococcus oralis | 3.0E-04 |  |  |  |
| Streptococcus cristatus | 3.0E-04 |  |  |  |
| Bacillus simplex | 3.0E-04 |  |  |  |
| Verminephrobacter eiseniae | 2.9E-04 |  |  |  |
| Altererythrobacter dongtanensis | 2.9E-04 |  |  |  |
| Acidovorax ebreus | 2.8E-04 |  |  |  |
| Citrobacter koseri | 2.8E-04 |  |  |  |
| [Haemophilus] ducreyi | 2.8E-04 |  |  |  |
| Pseudomonas stutzeri | 2.7E-04 |  |  |  |
| [Ruminococcus] torques | 2.7E-04 |  |  |  |
| Sphingomonas panacis | 2.5E-04 |  |  |  |
| Enterobacter cloacae | 2.5E-04 |  |  |  |
| Variovorax sp. PAMC 28711 | 2.4E-04 |  |  |  |
| Streptococcus salivarius | 2.4E-04 |  |  |  |
| Acidovorax avenae | 2.3E-04 |  |  |  |
| Streptococcus thermophilus | 2.3E-04 |  |  |  |
| Streptococcus sp. oral taxon 431 | 2.3E-04 |  |  |  |
| Hydrogenophaga sp. RAC07 | 2.2E-04 |  |  |  |
| Staphylococcus aureus | 2.2E-04 |  |  |  |
| Finegoldia magna | 2.2E-04 |  |  |  |
| Pseudomonas putida | 2.2E-04 |  |  |  |
| Ramlibacter tataouinensis | 2.2E-04 |  |  |  |
| Escherichia virus Lambda | 2.0E-04 |  |  |  |
| Polaromonas naphthalenivorans | 1.9E-04 |  |  |  |
| Streptococcus parasanguinis | 1.9E-04 |  |  |  |
| Bacillus licheniformis | 1.9E-04 |  |  |  |
| Delftia sp. Cs1-4 | 1.9E-04 |  |  |  |
| Cupriavidus metallidurans | 1.9E-04 |  |  |  |
| Bacillus thuringiensis | 1.8E-04 |  |  |  |
| Gemella sp. oral taxon 928 | 1.8E-04 |  |  |  |
| Choristoneura occidentalis granulovirus | 1.8E-04 |  |  |  |
| Salmonella enterica | 1.8E-04 |  |  |  |
| Neisseria elongata | 1.8E-04 |  |  |  |
| *(Continued)* | | | | |
| **SUpplementary Table 4 \|** Continued | | | | |
| **Taxonomic profile of bacteria and viruses species** | |  | **Taxonomic profile of viruses species only** | |
|  |  |  |  |  |
| **host NA depleted v1to10,000** (continued) |  |  |  |  |
| **Species** | **% Reads** |  | **Species** | **% Reads** |
| Rheinheimera sp. F8 | 1.7E-04 |  |  |  |
| Bradyrhizobium oligotrophicum | 1.7E-04 |  |  |  |
| Streptococcus suis | 1.7E-04 |  |  |  |
| Bradyrhizobium sp. CCGE-LA001 | 1.7E-04 |  |  |  |
